# Gymnemic Acids Inhibit Hyphal Growth and Virulence in *Candida albicans*

**DOI:** 10.1371/journal.pone.0074189

**Published:** 2013-09-11

**Authors:** Govindsamy Vediyappan, Vincent Dumontet, Franck Pelissier, Christophe d’Enfert

**Affiliations:** 1 Division of Biology, Kansas State University, Manhattan, Kansas, United States of America; 2 Institut Pasteur, Unité Biologie et Pathogénicité Fongiques, Département Génomes et Génétique, Paris, France; 3 INRA, USC2019, Paris, France; 4 CNRS, Institut de Chimie des Substances Naturelles, Centre de Recherche de Gif, Gif-sur-Yvette, France; New Jersey Medical School, Rutgers University, United States of America

## Abstract

*Candida albicans* is an opportunistic and polymorphic fungal pathogen that causes mucosal, disseminated and invasive infections in humans. Transition from the yeast form to the hyphal form is one of the key virulence factors in *C. albicans* contributing to macrophage evasion, tissue invasion and biofilm formation. Nontoxic small molecules that inhibit *C. albicans* yeast-to-hypha conversion and hyphal growth could represent a valuable source for understanding pathogenic fungal morphogenesis, identifying drug targets and serving as templates for the development of novel antifungal agents. Here, we have identified the triterpenoid saponin family of gymnemic acids (GAs) as inhibitor of *C. albicans* morphogenesis. GAs were isolated and purified from *Gymnema sylvestre* leaves, the Ayurvedic traditional medicinal plant used to treat diabetes. Purified GAs had no effect on the growth and viability of *C. albicans* yeast cells but inhibited its yeast-to-hypha conversion under several hypha-inducing conditions, including the presence of serum. Moreover, GAs promoted the conversion of *C. albicans* hyphae into yeast cells under hypha inducing conditions. They also inhibited conidial germination and hyphal growth of *Aspergillus* sp. Finally, GAs inhibited the formation of invasive hyphae from *C. albicans-*infected *Caenorhabditis elegans* worms and rescued them from killing by *C. albicans*. Hence, GAs could be useful for various antifungal applications due to their traditional use in herbal medicine.

## Introduction

Over the past decades, opportunistic fungal infections have gained increasing importance among nosocomial infections due to a growing number of patients who are immune-compromised or hospitalized with serious underlying diseases such as cancer, organ transplantation, non-transplant surgery or in neonatal intensive care units [1], [2], [3], [4]. A recent survey estimated that *Candida* spp. accounted for 88% of all nosocomial fungal infections in the U.S. of which 75% were invasive fungal infections costing the U.S. health care system around $3 billion annually [2].

*Candida albicans* is a commensal of human mucocutaneous surfaces such as the oral cavity, the gastrointestinal tract and the vaginal cavity. Yet, *C. albicans* causes superficial – oropharyngeal and vaginal – or hematogenously disseminated infections, when the host defense is compromised at the local or systemic level, respectively. Despite the availability of antifungal agents, the mortality associated to candidemia or invasive candidiasis remains high (30–50%) [2], [4]. Because *Candida* spp are eukaryotic fungal pathogens, developing antifungal therapeutics that are nontoxic to humans is challenging.

*C. albicans* cells exist in different morphological states (yeast, pseudohypha, hypha) and can undergo white-opaque phenotype switching in certain conditions. The ability to convert from yeast or pseudohyphal states to the hyphal growth state is critical for systemic infections, a premise that has been reinforced by the reduced virulence of various *C. albicans* mutants that are defective in hypha formation [5], [6]. Hyphal cells express cell wall adhesins and invade tissues thus causing deep-seated infection [7], [8], [9], [10]. The yeast-to-hypha conversion also plays a pivotal role in escaping from phagocytes [11], [12], [13]. Moreover, biofilm-mediated tolerance to various antifungal agents is well known in *C. albicans* and many hyphal growth-related genes are involved in biofilm formation [14], [15], [16].

*C. albicans* yeast-to-hypha transition occurs in response to various signals such as temperature (37°C), presence of serum, physiological CO_2_ concentration, neutral or alkaline pH, nutrient limitation and presence of amino acids [17], [18], [19], [20]. Several signaling pathways that respond to hypha-inducing cues have been identified in *C. albicans* including the cyclic AMP-protein kinase A (cAMP-PKA) pathway, a mitogen-activated protein kinase (MAPK) pathway, a cell cycle arrest pathway and a pH response pathway [19], [20], [21], [22]. The cAMP-PKA pathway is regarded as playing a pivotal role in *C. albicans* morphogenesis as it responds to a variety of hypha-inducing cues. Activation of the Cyr1 adenylate cyclase in response to these cues can be indirect following activation of the Ras1 and Gpa2 GTPases or direct in the case of CO_2_ or peptidoglycan in serum. Increased levels of cAMP result in the activation of the Tpk1 and Tpk2 catalytic subunits of PKA [20]. Several transcription factors that regulate the expression of hypha specific genes have been involved downstream of the cAMP-PKA pathway. In particular, Efg1 is a direct target of PKA and is considered the master regulator of the yeast-to-hypha transition. Other transcription factors such as Flo8, Tec1, Bcr1 and Ume6 act downstream of the cAMP-PKA pathway [20], [22]. Noticeably, over-expression of Ume6 is sufficient to drive hyphal formation in the absence of hypha-inducing cues and a functional cAMP-PKA-Efg1 pathway [23], [24]. Hyphal morphogenesis is also the subject of negative regulation by the general repressor Tup1 that acts in concert with the Nrg1 and Rfg1 DNA-binding proteins [25], [26]. Consequently, *C. albicans* mutants for the *TUP1* gene are constitutively filamentous [25].

Several small molecules that affect *C. albicans* morphogenesis have been identified [27]. Farnesol, fusel alcohols, *E-*nerolidol, farnesoic acid and tyrosol are produced by *C. albicans* and affect its morphogenesis (see references in review [27]). Farnesol, a quorum sensing sesquiterpene molecule, was shown to interfere with Ras1 signaling and to directly inhibit adenylate cyclase [28], [29]. Consequently, farnesol inhibition of the yeast-to-hypha transition can be rescued by addition of cAMP [28], [29]. *C. albicans* co-exists with various microorganisms in the host and the morphogenesis of *C. albicans* is affected by microbial secreted molecules such as 3-oxo-C12-acyl homoserine lactone, phenezine and pyocyanin produced by *Pseudomonas aeruginosa*, butyric acid produced by *Lactococcus* sp. and capric acid produced by *Saccharomyces boulardii* [27]. In addition, various lipid molecules, COX inhibitors, nisin Z lantibiotic peptide, histone deacetylase inhibitors, cell cycle inhibitors, calmodulin inhibitors, phospholipase D1, conjugated linoleic acid and undecylenic acid have been shown to affect *C. albicans* yeast-to-hypha transition through various pathways [27].

Small molecules that inhibit *C. albicans* yeast-to-hypha conversion but not its growth or viability could represent a valuable source for understanding pathogenic fungal morphogenesis and as templates for the development of novel antifungal agents. Here we report the isolation and identification of a family of plant-derived triterpenoid saponin compounds, the gymnemic acids (GAs), that inhibited *C. albicans* yeast-to-hypha transition under various hyphal inducing conditions, including in an animal (nematode) model of *Candida* infection. We also show that GAs trigger the conversion of *C. albicans* hyphae into yeast cells and inhibit conidial germination and hyphal growth of the filamentous fungal pathogen *Aspergillus fumigatus*. Thus, GAs can serve as probes for studying pathogenic fungal morphogenesis as well as templates for developing novel antifungal agents owing to their history of use in traditional medicine.

## Materials and Methods

### *C. albicans* Strains, Media and Growth Conditions

All fungal strains used in this study are listed in Table 1. *C. albicans* strain SC5314, an isolate from a patient with systemic candidiasis [30], was used for screening yeast-to-hypha inhibitors. Strains were routinely grown at 30°C on YPD medium (1% yeast extract, 2% peptone, 2% glucose). When necessary, YNB (0.67% yeast nitrogen base with amino acids, Difco) medium with 0.4% glucose was used. The impact of GAs on *C. albicans* yeast-to-hypha transition and hyphal growth was determined using several media. RPMI 1640 (Invitrogen, USA) medium buffered with 50 mM HEPES, pH 7.3, Lee’s medium, pH 6.8 [31], synthetic basal salts with N-acetyl-D-glucosamine (GlcNAc) [32], alkaline YPD medium (pH 9.0, [33]) or YPD plus 10% fetal bovine serum were used to induce hyphal formation at 37°C in liquid cultures. Yeast-peptone-sucrose (YPS) agar at 25°C (embedded condition [34]) was also used to determine the GAs effect on *C. albicans* hyphal growth. RPMI medium plus water agar mixture (1∶1) was used to monitor hyphal growth at 37°C in the presence or absence of GAs. Similar growth condition, except agar, was also used to determine the hyphal growth of *C. albicans* with or without GAs, 200 µM farnesol and/or 10 mM dibutyril-cAMP (db-cAMP, a membrane-permeable analog of cAMP). Sodium butyrate was used as a control for db-cAMP. Doxycycline (DOX; 20 µg/ml) was used to induce expression of *UME6* in *C. albicans* strain CEC1079. After 24 h incubation, mictrotiter plates containing the samples were viewed directly through an inverted Leica microscope. For other samples, Zeiss-Axioplan-2 microscope was used. Images were captured using a digital camera. To examine GAs effect on preformed hyphae, *C. albicans* SC5314 germ tubes were prepared by incubating yeast cells in buffered RPMI medium at 37°C with or without gentle shaking. After 4 h, GAs or solvent vehicle were added and the growth of germ tubes was continued for an additional 4–20 h. Amphotericin B (Sigma, USA) was used in some assays as positive antifungal control.

**Table 1:** Fungal strains used in this study.

Strains	Genotype	Reference
***Candida albicans***		
SC5314	Wild type	[30]
CEC161	*ura3*::λ*imm434/ura3*::λ*imm434 ARG4/arg4*::*hisG HIS1/his1*::*hisG*	[58]
CEC1365	*ura3*::λ*imm434/ura3*::λ*imm434 tup1*::*hisG/tup1*::*hisG*	[25]
CEC1366	*ura3*::λ*imm434/ura3*::λ*imm434 tup1*::*hisG/tup1*::(*hisG-URA3-hisG)*	[25]
CEC1049	*ura3*::λ*imm434/ura3*::λ*imm434 his1::hisG/HIS1 arg4::hisG/ARG4 ADH1/adh1::pADH1-CartTA::SAT1::*P*_TET_-CaGFP*	[56]
CEC1079	*ura3*::λ*imm434/ura3*::λ*imm434 his1::hisG/HIS1 arg4::hisG/ARG4 ADH1/adh1::pADH1-CartTA::SAT1::*P*_TET_-CaGFP RPS10/RPS10*::CIp10::P*_TET_-UME6*	[56]
***Aspergillus nidulans***		
MH1	*biA1*	R. B. Todd & GV’s lab collections, KSU
***Aspergillus fumigatus***		
FGSC#A1100	Wild-type	FGSC

### Screening for Inhibitors of *C. albicans* Yeast-to-hypha Conversion

A medicinal plant-derived library of semi-purified compounds was obtained through Laksbiotec (Pvt.), India. Briefly, the plant extracts from 50 different plants covering about 30 families were fractionated through silica gel (grade 62, 60–200 mesh) columns and vacuum dried. Compounds were dissolved in DMSO (1 mg/ml) and small aliquots (5–10 µl/100 µl) were used for assays in 96-well plates. *C. albicans* SC5314 strain was used to screen for compounds that inhibit yeast-to-hypha conversion and hyphal growth in RPMI medium at 37°C. The main criteria for isolating inhibitors (“hits”) of *C. albicans* yeast-to-hypha conversion from these plant derived sources included: (i) compounds should inhibit the yeast-to-hypha transition under various hypha inducing growth conditions and (ii) compounds should be non-toxic to *C. albicans* cells. Primary screening results were confirmed by secondary assays with additional growth conditions including YPD medium with 10% serum or RPMI agar medium. Although we identified 6 different hits, one potential plant source (*G. sylvestre*, GS) was selected for isolation and identification of active principle(s). The effect of bioactive fractions on *C. albicans* growth was determined in YPD broth as well as RPMI medium using microtiter wells in the presence or absence of fraction and measuring the absorbance at OD_630_. Aliquots of cells from control and compound-treated wells were serially diluted ten fold and spot tested on YPD agar plates. After incubation at 30°C for 16 h, the growth of colonies was recorded.

### Purification of Active Principle(s) and Biological Assay

Bioassay-guided purification of GS leaf extract was conducted mainly as described by Liu *et. al*. [35] with the following modifications. Briefly, *G. sylvestre* leaf powder (200 g, obtained from Laksbiotec Pvt., India) was extracted with 75% ethanol and vacuum dried. The brownish residue (100 g) was further extracted sequentially with petroleum ether and methanol to remove fatty acid components. The methanol extract (82 g) was treated with activated charcoal and particle free methanol extract was vacuum dried (46 g). The brownish crystal-like *G. sylvestre* crude sample (800 mg out of the 46 g) was dissolved in MeOH and fractionated by flash chromatography on a reverse phase column (RediSep) using a Companion Combiflash (A gradient mobile phase of acetonitrile-water with 0.1% formic acid was used as follows: 20% acetonitrile in water to 100% acetonitrile in 30 minutes, at the flow rate of 60 ml/minute throughout the run). Fractions were assayed for their inhibitory activity against *C. albicans* yeast-to-hypha conversion and the most active fraction (75 mg) was fractionated on preparative HPLC column (Hypersil HS C18, 250×19 mm, i. d., 5 µm) using Waters 2995 PDA (PhotoDiode Array), Waters 2424 ELS (Evaporative Light Scattering) detectors and Waters 600 Pump system. A gradient mobile phase of MeOH-water with 0.1% formic acid was used as follows: 20% MeOH in water to 100% MeOH in 30 minutes, followed by 100% MeOH for 10 minutes at the flow rate of 17 ml/minute throughout the run. Fractions were assayed for biological activity and only the most active fraction (37 mg) was collected, and refractionated by employing the optimized protocol [semi-preparative HPLC column (Sunfire C18, 250×10 mm, i. d., 5 µm) using Waters 2995 PDA (PhotoDiode Array), Waters 2424 ELS (Evaporative Light Scattering) detectors and Water 600 Pump system]. An isocratic mobile phase of MeOH-water with 0.1% formic acid was used to purify the fractions as follows: 35% MeOH in water at the flow rate of 6.5 ml/minute throughout the run. Ten fractions based on peak detection (ELSD detector) were collected using an automated fraction collector. Individual fractions F1–F10 were vacuum dried. In order to verify the purity of the compounds for subsequent identification, samples were re-tested on analytical HPLC (Sunfire C18, 250×4.6 i. d., 5 µm; Waters Alliance 2695, PDA 996, ELSD 2420, ZQ 2000) and fractions that showed single peaks, thus likely to contain pure compounds, were subjected to further analyses. Based on analytical HPLC and MS evaluations, four fractions (F2, F5, F7 and F8) were selected. To determine the structures of the purified compounds in fractions F2, F5, F7 and F8, HRESIMS, ^1^H and ^13^C NMR were used. ESIMS and HRESIMS were run on an ESI-TOF spectrometer (LCT; Waters®). The NMR spectra were recorded in C_5_D_5_N on an Avance 600 Bruker spectrometer equipped with a PATXI 1.7 mm probe operating at 599.46 and 150.75 MHz, respectively. ^1^H chemical shifts were referenced relative to central peak of C_5_D_5_N at 7.58 ppm and ^13^C chemical shifts to central peak of C_5_D_5_N at 135.91 ppm. Based on the spectroscopic data and their similarity to the GAs reported in the literature [35], [36], [37], [38], purified GAs were identified. Fraction F2 led to 2.4 mg of GA-IV (**2**), F5 to 1.7 mg of GA-III (**1**), F7 to 1.9 mg of GA-XIV (**4**), and F8 to 3.7 mg of GA-XIII (**3**). GA-VIII and GA-IX were also purified through this procedure. Yet, limited quantities precluded their further analysis. Purified compounds were solubilized in 70% methanol and used in biological assays. Mixtures of 4 GA species (GA-III, IV, XIII and XIV (at equal proportion, 10 µg each) 40 µg/ml [∼52 µM]) were used in this study and referred as GAs throughout.

### *Caenorhabditis elegans* Host Model for *C. albicans* Hyphal Growth and Virulence Inhibition

To test the impact of GAs on *C. albicans* virulence, a non-mammalian host model *Caenorhabditis elegans* (wild type) was used as reported [39], [40] with slight modifications. Briefly, L2 stage larvae were fed on a *C. albicans* SC5314 yeast lawn. After collecting the larvae and washing off yeast cells with PBS buffer, an aliquot of larvae was added to microtiter wells containing buffered RPMI or YNB medium with or without GAs. As a positive antifungal control, amphotericin-B (AMB, 1 µg/ml) was included in parallel assays. The assay plate was placed in a plastic box lined with moisture paper and the whole box was incubated at 30°C for 2–4 days with gentle shaking. Triplicate wells, about 10–15 worms/well, were used for each assay and the assay was repeated three times. Worms were monitored each day using an inverted microscope and the results were recorded with a digital camera attached to microscope. Percent live or dead worms due to *C. albicans* infection in the presence or absence of GAs were calculated from data collected after 4 days of incubation. To visualize *C. albicans* yeast cells in worms, worms were immobilized in molten water agar (0.2%) containing 0.1% sodium azide, mounted on glass slide and viewed by confocal microscopy.

### Cytotoxicity and Hemolytic Activity Assays with Gas

Kidney cell line, BS-C-1, derived from African green monkey [41], [42] was obtained from American Type Culture Collection (ATCC, CCL-26). BS-C-1 cells and human intestinal epithelial cells (Int-407) [43] were maintained on RPMI 1640 with 10% serum under 5% CO_2_ at 37°C. Two-day old monolayers of cells in 96-well microtiter plates were treated with GAs (40 µg/ml) or #194 (300 µg/ml) for 24 h in the growth medium. Controls including no treatment and solvents (DMSO or 75% methanol both <5%) treated cells were also included in parallel. Viability of cells was determined by spectrometric methods of live dead viability assay (Promega corporation, WI) as described by the manufacturer and observing cells for rounding and detachments using microscopy.

Hemolytic assay was performed on tryptic soy agar plate containing human red blood cells (hRBC, 5%). Different fractions containing GAs were diluted in PBS and spotted on hRBC-agar. Positive controls including actively growing *Staphylococcus aureus* cells (2 µl) or PBS containing Triton X-100 (1%) were also spotted on the blood agar medium. Plates were incubated for 24–48 h at 37°C and hemolytic activity (halos around the spots) were recorded.

## Results and Discussion

### *Gymnema sylvestre* Fractions Inhibit *C. albicans* Yeast-to-hypha Conversion

To identify inhibitors of *C. albicans* yeast-to-hypha conversion we used a small collection of medicinal plant-derived compounds. Plants are constantly exposed to various pathogens (viruses, bacteria and fungi) and have built-in defense mechanisms, notably secondary metabolites [44]. As many of the plant pathogenic fungi enter into the plant cells via hypha-dependent penetration structures, we reasoned that plants should produce compounds that can limit/inhibit hyphal growth of the invading fungal pathogens and could represent a useful resource for the identification of inhibitors of yeast-to-hypha transition and hyphal growth in *C. albicans*. We focused on medicinal plant sources as they are used in traditional medicines to treat various ailments thus supporting their safe use in humans.

**Figure 1:**
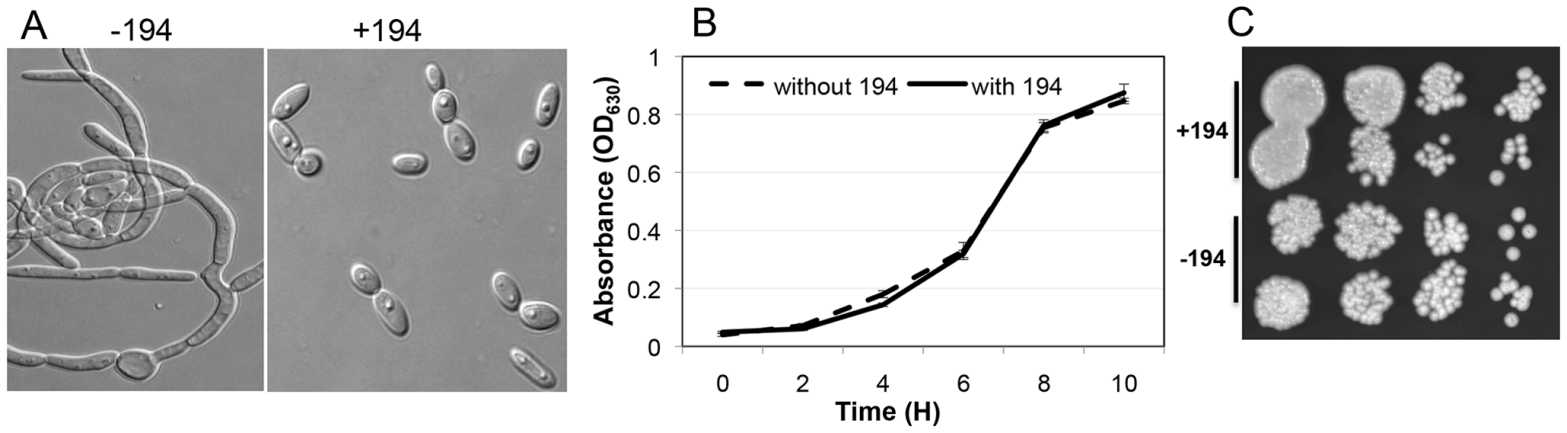
Effect of a *Gymnema sylvestre* fraction (#194) on *Candida albicans* yeast-to-hypha conversion, growth and viability. (A) Stationary-phase *C. albicans* yeast cells grown in YNB medium were resuspended (1×10^5^ cfu/ml) in RPMI 1640 medium +50 mM glucose (buffered with HEPES 50 mM, pH 7.3) containing equal volume of DMSO (-194) or in the presence of fraction #194 and incubated in microtiter plates at 37°C with gentle shaking for 16 h. Cells were viewed under microscope and photographed. (B) Growth of *C. albicans* in the presence or absence of fraction #194 (but with equal volume of DMSO). Yeast cells were incubated in YPD liquid medium at 30°C in microtiter wells without shaking for the indicated times and growth of cells was determined by measuring absorbance (OD_630_). Experiments were repeated at least twice each with triplicates. Error bars indicate standard deviations (SD). (C) Viability of cells exposed to vehicle control or fraction #194 in RPMI medium at 37°C were determined by removing aliquots of cell suspensions at t = 8 h of growth, vortexing for 30 seconds at top speed and diluting them ten fold serially before spotting 5 µl on YPD agar plates. Plates were incubated at 30°C for 16 h and then photographed.

In order to improve and enrich the detection of active principles, plant extracts were fractionated on conventional silica gel columns and concentrated fractions were used for initial screening. Using RPMI medium at 37°C as hypha-inducing growth condition and a microtiter plate-based assay, we screened about 600 semi-purified fractions derived from 50 plants. A set of fractions derived from the plant *Gymnema sylvestre* consistently showed strong inhibitory activity against *C. albicans* yeast-to-hypha conversion. *G. sylvestre* (Retz.) R. Br. (Asclepiadaceae family) is extensively used in Ayurveda traditional medicine in India particularly for the management of diabetes and is well known for its antisweet properties [45], [46], [47]. Results presented in Fig. 1A showed that one *G. sylvestre* fraction, #194 (ca. 50 µg/ml), inhibited the conversion to germ tubes of more than 90% of the yeast cells even after 24 h of incubation while untreated cells had all undergone the yeast-to-hypha transition. Exposure to #194 did not affect *C. albicans* yeast growth in YPD medium (Fig. 1B). Moreover, *C. albicans* cells that had been exposed to #194 under hypha-inducing conditions (RPMI, 37°C) or yeast-promoting conditions did not show reduced viability when transferred to YPD medium lacking #194 (Fig. 1C and data not shown). These results suggested that *G. sylvestre* fraction #194 was nontoxic to *C. albicans* and contained one or more inhibitors of *C. albicans* morphogenesis.

### Bioassay-guided Purification of *Gymnema sylvestre* Leaf Extracts and Identification of Active Principles

**Figure 2:**
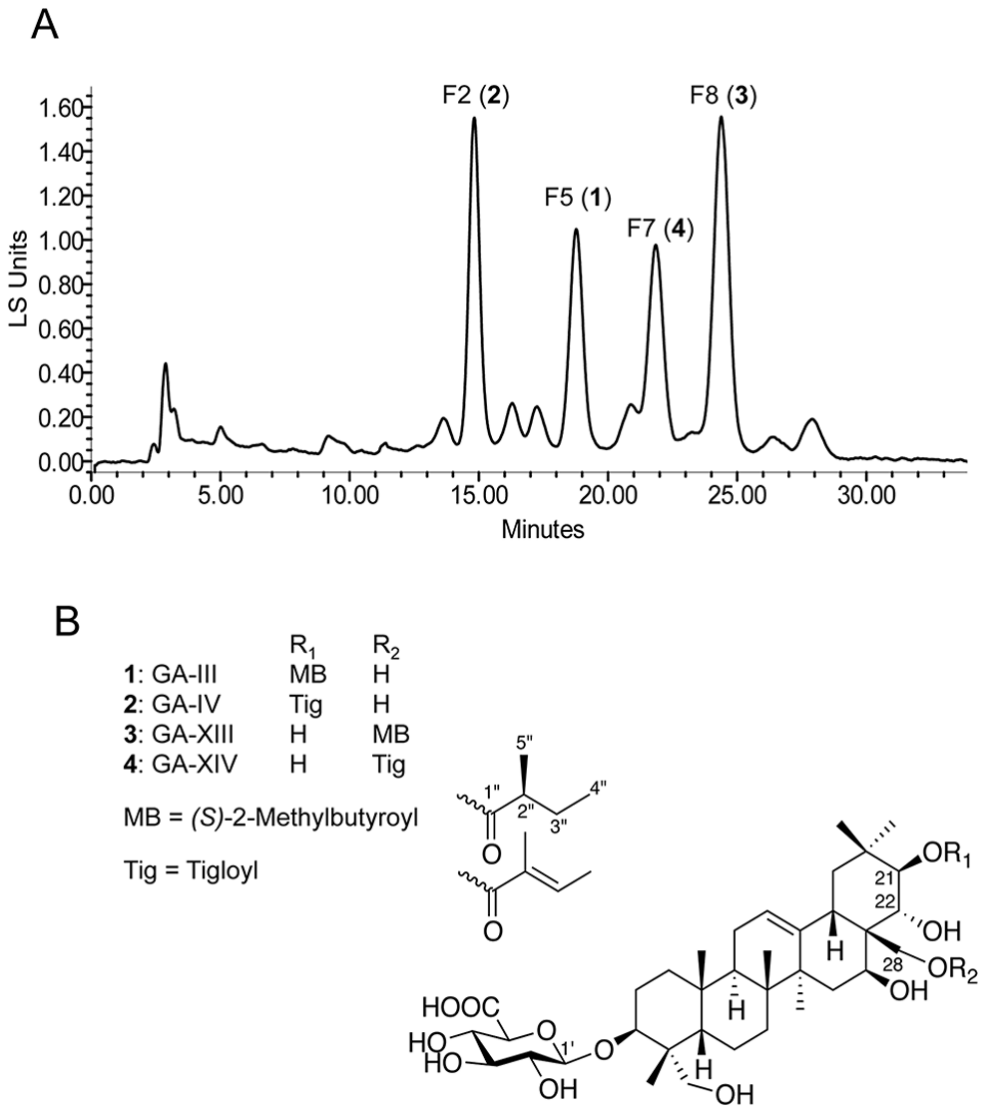
Purification and identification of gymnemic acids (GAs). (A) Solvent extracted and semi-purified GAs were fractionated on preparative HPLC (Sunfire C_18_ 5 µm, 250×10 mm) using an isocratic mobile phase (see “Materials and Methods” for details). Fractions with major peaks were collected using an automated fraction collector, vacuum dried and assayed for inhibition of *C. albicans* yeast-to-hypha conversion. Individual fractions (F2, F5, F7 and F8) were evaluated for purity and molecular weight analyses using analytical HPLC-ELSD-DAD-MS, ESIMS, HRESIMS, ^1^H NMR and ^13^C NMR (see Figures S1–S20). (B) The four gymnemic acids (**1–4**) were identified using mass and NMR data (see Figures S1–S20 for details of GA species) according to Liu *et. al*. [35] and Yoshikawa *et. al*. [36], [37], [38]. The general structure of GA, methylbutyroyl and tigloyl are shown.

*G. sylvestre* is known to contain a number of phytochemicals such as gymnemic acids (GAs), a family of triterpenoid compounds [35], [36], [37], [38]. Yet, the activity of these phytochemicals towards fungi has not been studied. In order to isolate and identify inhibitors of *C. albicans* morphogenesis from *G. sylvestre*, a bioassay-guided purification was undertaken. Dried leaf powder of *G. sylvestre* was extracted with 75% ethanol and vacuum dried. After multiple steps of solvent extractions, the active crude extract was fractionated by reverse phase chromatography (see Materials and Methods for details). A representative chromatogram of final preparative HPLC for a *G. sylvestre* semi-purified extract is shown in Fig. 2A. Samples showing distinct peaks were collected individually and assayed for inhibition of *C. albicans* yeast-to-hypha conversion. Among these samples, four major fractions (F2, F5, F7 and F8; Fig. 2A) inhibited *C. albicans* yeast-to-hypha transition. The remaining fractions also showed different levels of inhibitory activity but were more complex in composition and were not characterized further in this study.

Further fractionation of F2, F5, F7 and F8 indicated that they contained pure compounds designated **2**, **1**, **4**, **3**, respectively. Characterization of these compounds by HPLC-ELSD-DAD-MS, ESIMS, HRESIMS, ^1^H NMR and ^13^C NMR showed that they corresponded to GA-IV, GA-III, GA-XIV and GA-XIII, respectively (Fig. 2B and Figures S1, S2, S3, S4, S5, S6, S7, S8, S9, S10, S11, S12, S13, S14, S15, S16, S17, S18, S19, S20) [48]. Indeed, the NMR data of these four compounds showed chemical shifts characteristic of the triterpenoid skeleton of GAs [35], [36], [37], [38]. HRESIMS of compounds **1** and **3** indicated a pseudo molecular ion peak at m/z 767.4575 [M+H]^+^ and m/z 767.4587 [M+H]^+^, respectively, giving for both the molecular formula C_41_H_66_O_13_ (calcd. 766.4503). Mass and NMR data of **1** and **3** correspond either to GA-III and GA-XIII [35], [37], [38]. The chemical shifts at δ_C-1″_ 176.9 or 176.5 ppm, δ_C-3″_ 27.6 or 27.4 and δ_C-4″_ 17.5 or 17.6 ppm, for **1** and **3**, respectively, confirmed the presence of a (*S*)-2-methylbutyroyl group. Occasionally, the sugar related resonance signals (*e. g.* **1**) was not readily detectable and this could be due to different relaxing times for sugar and saponin. The chemical shifts at δ_C-21_ 79.5, δ_C-22_ 71.7 and δ_C-28_ 58.7 ppm for **1** were in accordance with those observed for GA-III (the ester linkage shifts C-21 to the downfield region), while the chemical shifts at δ_C-21_ 77.2, δ_C-22_ 74.4 and δ_C-28_ 62.6 ppm for **3** were in accordance with the chemical shifts observed for GA-XIII (the ester linkage shifts C-28 to the downfield region). HRESIMS of compounds **2** and **4** indicated a pseudo molecular ion peak at m/z 787.4240 [M+Na]^+^ and m/z 765.4429 [M+H]^+^, respectively, giving for both the molecular formula C_41_H_64_O_13_ (calcd. 764.4347). Mass and NMR data of **2** and **4** correspond either to GA-IV and GA-XIV [35], [37], [38]. The chemical shifts at δ_C-1″_ 168.6 or 168.3 ppm, δ_C-2″_ 130.1 or 129.6 ppm, for **2** and **4**, respectively, and δ_C-3″_ 136.4 ppm and δ_C-4″_ 14.5 ppm, for both **2** and **4**, confirmed the presence of a tigloyl group. The same shifts values of C-21or C-28 occurred for **2** and **4** compared to **1** and **3**. Compound **2** is thus assigned to GA-IV and compound **4** to GA-XIV (Figures S1, S2, S3, S4, S5, S6, S7, S8, S9, S10, S11, S12, S13, S14, S15, S16, S17, S18, S19, S20).

As mentioned above, GAs form a family of triterpenoid saponin compounds whose biological activities against fungi were not known [35], [36], [37], [38], [47], [49]. A recent study has reported the identification of several plant-derived saponin compounds that inhibited *C. albicans* growth using the *Caenorhabditis elegans* infection model as a screening assay [40]. Yet, GAs differed from these saponins through their structure and, most importantly, their specific inhibition of hyphal growth.

### Biological Activity of GAs on *C. albicans* Yeast-to-hypha Transition

**Figure 3:**
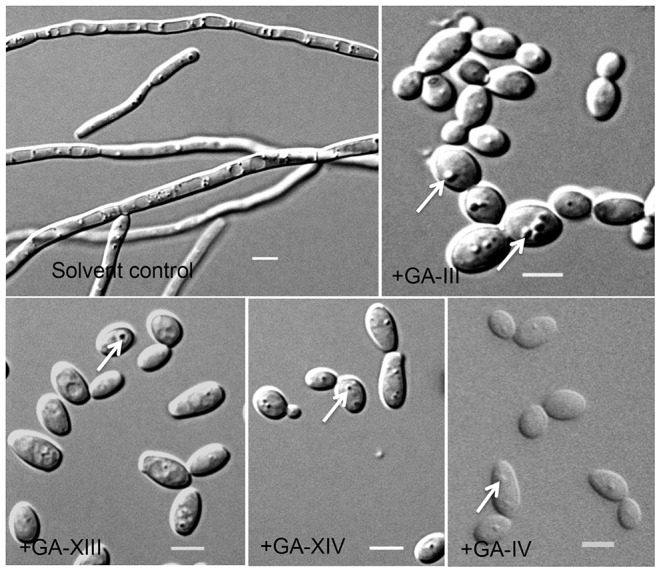
Inhibition of *C. albicans* yeast-to-hypha transition by individual GAs. *C. albicans* yeast cells were incubated in hyphae inducing medium (RPMI) in microtiter wells without shaking at 37°C with the indicated GA for 16 h. Each GA was solubilized in 75% methanol and added to the yeast cell suspension at final concentrations of 60 µg/ml. The final concentration of solvent was <5%. Cells were monitored under microscope using 10x×63x objective (Zeiss) and images were recorded. Solvent control contains equal volume of 75% methanol. Arrows show vesicle like structures in yeast cells. Scale bars = 5 µm.

Results presented in Fig. 3 showed that pure GA-III (**1**), GA-IV (**2**), GA-XIII (**3**) and GA-XIV (**4**) inhibited equally well *C. albicans* yeast-to-hypha transition when used at a 78 µM (60 µg/ml) concentration. Yet, because commercially purified GAs are not available and the purification strategies were tedious yielding limited quantities of individual GAs, a GA- mixture (GA-III, -IV, -XIII and -XIV, 40 µg/ml) was used in the assays described below. We selected a concentration that gave maximum inhibitory activity of *C. albicans* yeast-to-hypha conversion.

**Figure 4:**
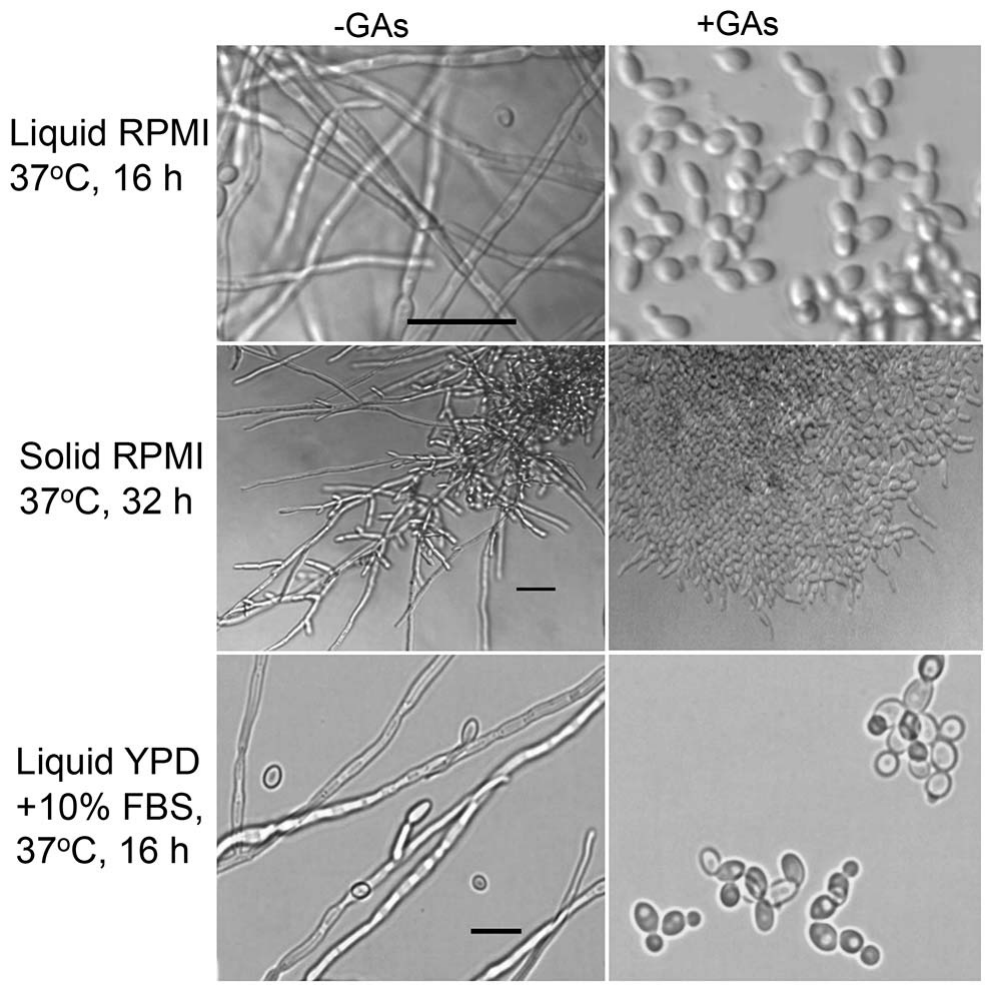
Gymnemic acids inhibit hyphal formation and extension by *C. albicans*. Effect of the addition of a mixture of GAs (GAs, 40 µg/ml) on the yeast-to-hypha conversion and filamentation induced by liquid, solid RPMI or in liquid YPD in the presence of 10% fetal bovine serum at 37°C in microtiter wells. Scale bars = 25 µm.

**Figure 5:**
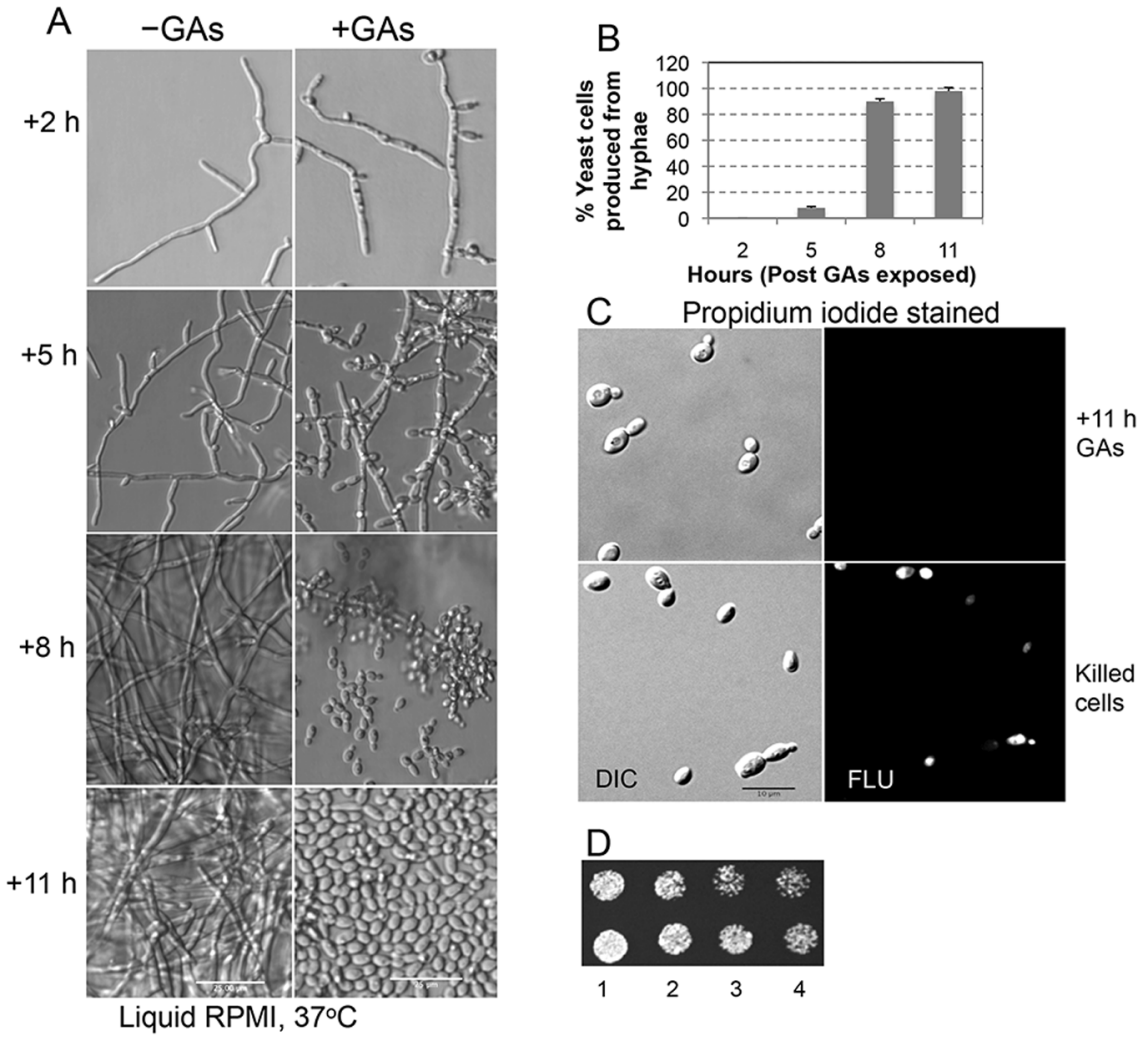
GAs-mediated conversion of *C. albicans* hyphae into yeast cells. (A) Four hours old hyphae of *C. albicans* were incubated in hyphal growth promoting medium (RPMI) at 37°C with or without GAs in microtiter wells under static condition. At the indicated post incubation time intervals (+2, +5, +8 and +11 h), conversion of hyphae into yeasts was monitored using an inverted microscope. Scale bars = 25 µm. (B) Percentage of released yeast cells from hyphae due to GAs exposure, at least from 3 different wells, were counted at each time points. Error bars indicate standard deviation. (C) Live/dead assay of yeast cells using propidium iodide (PI) stain was performed with the cells generated from GAs-exposed hyphae. An aliquot of cells from +11 h sample was stained with PI and viewed under fluorescence (FLU) microscope (Zeiss) with red filter. Corresponding DIC images were also recorded. As a positive control, yeast cells were killed by exposing them to 100% ethanol for 5 minutes and washed twice with sterile water to remove ethanol. Cells were stained with PI in parallel with test samples. Scale bar = 10 µm. (D) Viability of cells from 11 h post GAs-treated samples (duplicates) were two fold-serially diluted and 5 µl from each dilution (1 to 4) were spotted on YPD agar plate. Growth of cells was assessed after 16 h incubation at 30°C. Sparse growth of colonies can be seen from the 4^th^ diluted samples.

Results presented in Fig. 4 showed that GAs had inhibitory activity of *C. albicans* yeast-to-hypha conversion under several hypha-inducing conditions such as liquid or solid RPMI and YPD medium containing 10% fetal bovine serum at 37°C. In contrast, purified GAs did not affect the growth or viability of yeast cells as determined by plating GAs exposed cells on YPD (data not shown, and see results in Fig. 5), consistent with earlier observations with fraction #194 (Fig. 1). Additional hypha-inducing conditions (Lee’s medium, basal salts with GlcNAc, alkaline YPD at 37°C and yeast-peptone-sucrose (YPS) agar at 25°C (embedded condition [34])) were also used to examine GA’s inhibitory effect on *C. albicans* yeast-to-hypha transition and hyphal growth. GAs inhibited yeast-to-hyphal transition and hyphal growth in Lee’s medium, GlcNAc containing medium (Figure S21) and alkaline medium (data not included). However, GAs did not block *C. albicans* hyphal growth in embedded condition (data not shown) suggesting that GAs may not act on the contact-dependent *CZF1* pathway [34]. Future studies will determine the impacts of GAs on *C. albicans* hyphal growth in embedded YPS with various ratios of O_2_/CO_2_ and the expression of *CZF1* in embedded YPS with or without GAs. Thus, except in embedded YPS, GAs inhibited *C. albicans* yeast-to-hypha transition in all hypha inducing conditions, precluding the possibility that they depleted hypha promoting factors from various media.

Incubation of GAs with 4 h old actively growing germ tubes under hypha-inducing conditions prevented their hyphal extension and triggered the production of yeast cells from hyphae (Fig. 5). Exposure of germ tubes to GAs triggered morphological changes as early as 2 h along the hyphae (*e. g*. initiation of budding and formation of vesicular structures in them, see Fig. 5A, +2 h). Production of yeast cells started 5 h post exposure to GAs and reached a maximum level at 11 h (Fig. 5A and B). These yeast cells were viable as determined by live-dead staining with propidium iodide and growth assessment on YPD agar (Fig. 5C and D). The budding and release of yeast cells from GAs exposed hyphae increased with agitation (data not shown).

**Figure 6:**
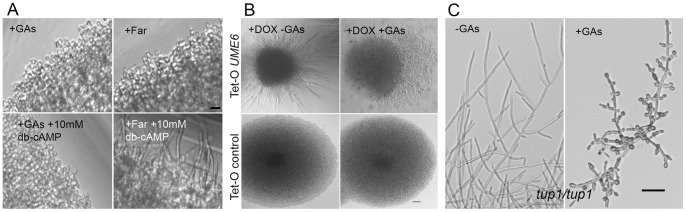
Effect of GAs on various hyphal growth regulatory pathways. (A) cAMP cannot rescue GAs mediated hyphal growth inhibition. *C. albicans* yeast cells were incubated in buffered RPMI medium +50 mM glucose at 37°C in 96 well plates without shaking. GAs or farnesol (Far) was added to the yeast cell suspension separately at a final concentration of 40 µg/ml or 200 µM, respectively. In addition to these hyphal growth inhibitors, db-cAMP was premixed at 10 mM (final concentration) prior to initiating the growth. After 24 h incubation, mictrotiter plates containing the samples were viewed directly through inverted microscope (Leica) with 10x ocular×20x objective lenses. Images were captured using a digital camera. To show the hypha or yeast growth distinctly, the peripheries of the growing area are presented. Scale bar = 10 µm. (B) GAs inhibit Ume6-induced filamentation. *C. albicans* expressing a Tet-inducible *UME6* was incubated in a yeast promoting growth medium (YPD at 30°C) in the presence of doxycycline (DOX) and with or without GAs. As a control, strain CEC1049 (Table 1) lacking the P*_TET_*-*UME6* construct was used. Individual colonies grown on YPD agar in microwells were visualized and images were recorded with a digital camera. Scale bar = 25 µm. (C) GAs inhibit the constitutive hyphal growth of *tup1*Δ*/*Δ mutant and induces bud formation. *tup1*Δ/Δ cells were grown in yeast growth supporting medium (liquid YPD at 30°C) in the presence or absence of GAs for 16 h. Scale bar = 25 µm. A *tup1*Δ/Δ*-URA3* marker complemented strain was also included in parallel assays and similar results were found (pictures not shown).

In *C. albicans* a variety of mutations or compounds have been shown to bypass the requirement of morphogenesis for hypha-inducing cues and signaling components. Examples include dibutiryl-cAMP (db-cAMP) that allows bypassing hypha-inducing cues by directly triggering activation of PKA [28], [29], over-expression of *UME6* that triggers hyphal differentiation independently of a functional cAMP-PKA-Efg1 pathway [23], [24], [50] and deletion of *TUP1* that leads to constitutive hyphal growth under many growth conditions [25]. Hence, we tested whether GAs could impact filamentation of a wild-type strain grown in the presence of db-cAMP or *C. albicans* mutants overexpressing *UME6* or lacking *TUP1.* Results shown in Fig. 6A revealed that GAs impaired hyphal growth in the presence of db-cAMP. Moreover, GAs impaired hyphal growth of the *C. albicans UME6-*overexpression strain and *tup1*Δ*/*Δ mutant, and promoted the release of yeast cells from hyphae formed by these strains (Fig. 6B and C).

Taken together, these results indicated that GAs were stable and potent inhibitors of the initiation and maintenance of hyphal growth in *C. albicans* and had the ability to reprogram *C. albicans* polarized hyphal growth into yeast growth. Gymnema derived extracts or compounds have multiple but unexplained pharmacological activities such as antisweet, antihyperglycemic, glucose uptake inhibitory, antiobesity, antiviral, gut glycosidase inhibitory activities [45], [46], [47]. Our results add another, previously unrecognized activity, to Gymnema derived extracts and, more specifically, gymnemic acids. Moreover, our results indicate that GAs have the ability to prevent the yeast-to-hypha transition and promote the hypha-to-yeast transition under a variety of conditions that normally promote hyphal growth (chemical activation of PKA, activation of *UME6* and derepression through inactivation of *TUP1*), suggesting that they target a pathway whose functionality is necessary for hyphal morphogenesis under a variety of (if not all) inducing conditions. Notably, db-cAMP did not relieve GAs-dependent inhibition of hyphal morphogenesis in contrast to what was observed for farnesol ([28], [29] and Fig. 6A), suggesting that GAs do not act by inhibiting adenylate cyclase.

### Biological Activity of GAs on *Aspergillus fumigatus* Conidial Germination and Hyphal Growth

**Figure 7:**
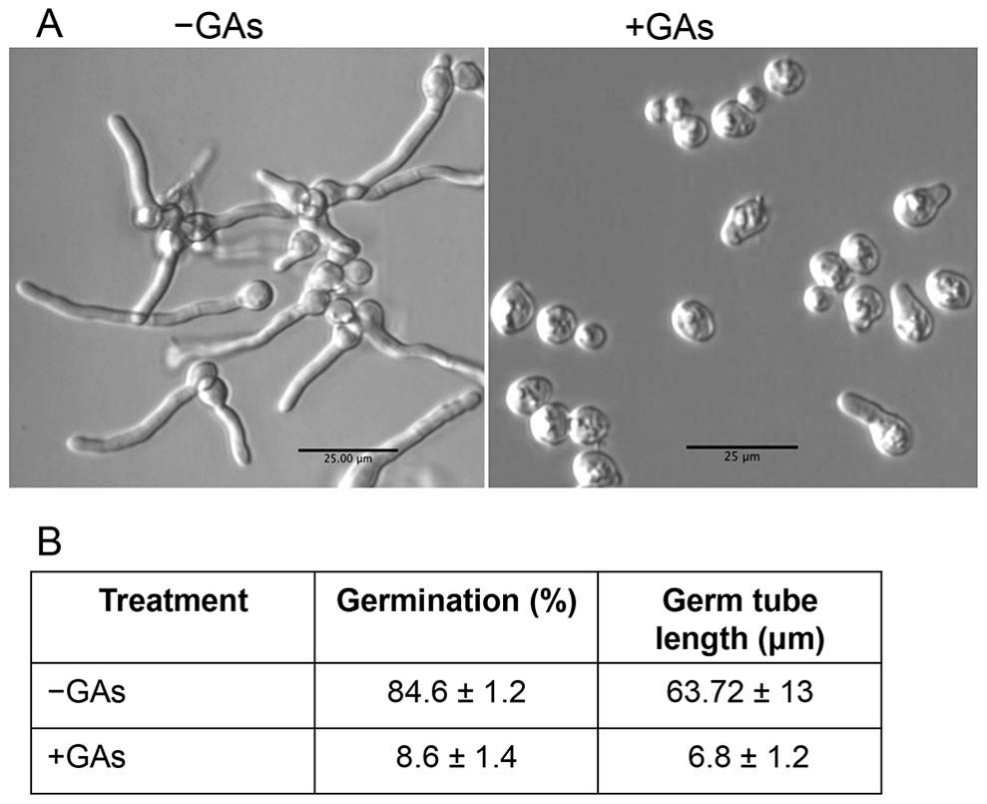
Effect of GAs on germination of *Aspergillus fumigatus* spores. (A) A conidiospore suspension was incubated in RPMI medium at 37°C with (40 µg/ml) or without GAs for 15 h under static condition in microtiter wells. Percentage of germination was calculated at least from nine different fields from triplicate wells. A spore is considered germinated when the length of the germ tube is twice or more the size of a spore. Scale bars = 25 µm. (B) Table showing the impact of GAs on *A. fumigatus* spore germination and germ tube lengths. The lengths of germ tubes were measured by using µScope software (µScope Essential) and shown ± SD.

To further examine the hyphal growth inhibitory activity of GAs, germination and hyphal growth of the filamentous pathogenic fungus *Aspergillus fumigatus* was tested with (40 µg/ml) and without GAs as above. Results shown in Fig. 7 indicated that GAs impaired germination and hyphal growth of *A. fumigatus*. Similar results were obtained with *Aspergillus nidulans* (data not shown). Note that these fungi do not form yeast and hence their continuous hyphal growth was severely affected.

### GAs Rescue *Caenorhabditis elegans* Survival from Killing by *C. albicans* Hyphae

**Figure 8:**
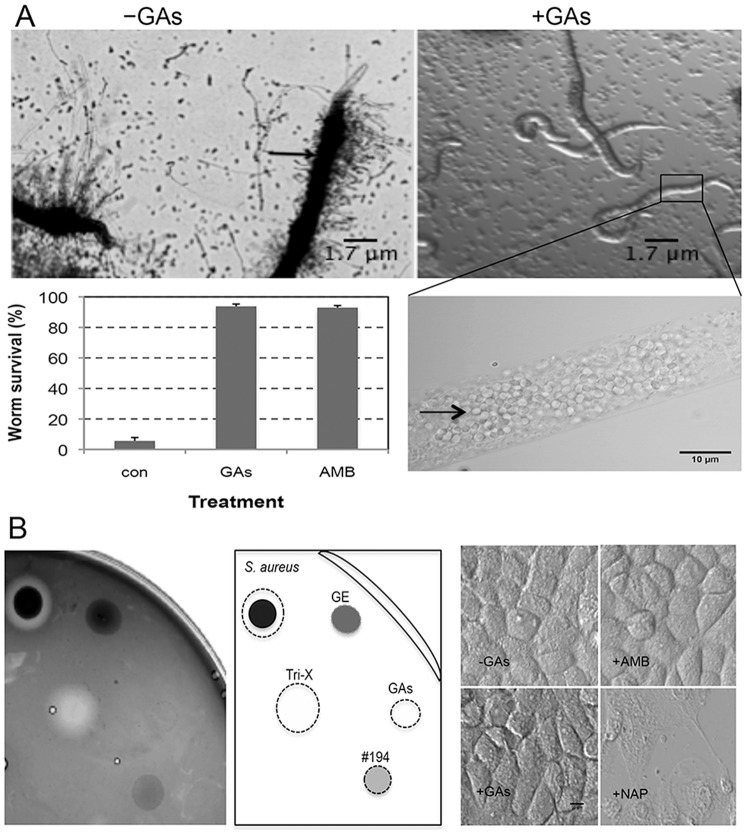
Effect of GAs on *C. albicans* infection in *Caenorhabditis elegans* and mammalian cells. (A) Rescue of *C. albicans* infected *C. elegans* from death by GAs. Larvae of *C. elegans* fed with yeast cells of *C. albicans* were incubated in RPMI medium with or without GAs (40 µg/ml) in a 96 well microtiter plate and incubated at 30°C for 2–4 days. Arrow in the top left panel (-GAs) shows the growth of *C. albicans* hyphae from the dead worms while addition of GAs (+GAs) prevent growth of hyphae from the worms’ body and hence worms survival (top right panel). Small round structures in the background are *C. albicans* yeast cells. Inset of *C. elegans* from GAs treated well shows confocal microscopic image of *C. elegans* containing *C. albicans* yeast cells in the worm’s gut (arrow). Scale bar (inset) = 10 µm. Bar graph at lower left panel indicates the % worms surviving after 4 days of exposure to GAs or to AMB. Survival of worms was determined by their movements and absence of hyphal growth from worms using microscope. Error bars indicate SD from the averages of 3 independent experiments. (B) GAs are non hemolytic and nontoxic to mammalian cells. Hemolytic assay was performed on tryptic soy agar plate containing human red blood cells (hRBC, 5%) (left side). A diagrammatic representation with sample identity is shown on the right side. Different fractions containing GAs [*G. sylvestre* extract, GE 1 mg/ml, 4 µl; fraction #194 (4 µl); and purified GAs (40 µg/ml, 4 µl)] were diluted in PBS and spotted on hRBC-agar. Positive controls including actively growing *Staphylococcus aureus* cells (2 µl) or PBS containing Triton X-100 (1%) (Tri-X) were also spotted on the blood agar medium as controls. Plates were incubated for 24–48 h at 37°C and the results were recorded by image capture. White clear halos around spots indicate hemolytic activity. GAs are not toxic to mammalian kidney epithelial cells (far right sector). Napthaquinone (NAP, 50 µg/ml) killed the kidney epithelial cells whereas solvent control (-GAs), amphotericin B (+AMB) or test compounds (+GAs; 40 µg/ml) did not. Scale bar = 10 µm.

We next tested if GAs could affect *C. albicans* virulence in a nematode model of Candida infection, an alternative to mammalian host models [39]. This assay allows simultaneous assessment of a compound’s toxicity and antifungal efficacy towards *C. albicans*. While the yeast growth form of *C. albicans* is non destructive to *Caenorhabditis elegans* and hence non-lethal to it [51], piercing through the nematode’s cuticle by the hyphal form of growth kills the worm [39], [51]. Worms fed with *C. albicans* yeast cells were incubated in buffered RPMI medium in the presence or absence of GAs. We found that most of the nematodes (>90%) survived from the lethal effect of *C. albicans* hyphal growth in the presence of GAs (Fig. 8A). GAs-treated *C. elegans* harbored *C. albicans* yeast cells in the gut, suggesting that GAs treatment inhibited the yeast-to-hypha conversion and hyphal growth from the nematodes and therefore prevented *C. albicans*-mediated killing of the worms (Fig. 8A, right panels inset and arrow). In contrast, a majority of the worms in control wells (without GAs) died mainly due to the invasive growth of *C. albicans* hyphae from the worm’s body (Fig. 8A, left panel and arrow). These results suggested that GAs are nontoxic to worms and could prevent invasive hyphal growth of *C. albicans* emerging from worms.

### GAs are Non Hemolytic and Nontoxic to Mammalian Cells

Although GAs are terpenoid saponins that are not known to affect cellular membranes, steroidal saponins can affect cellular membranes and cause cellular leakage [52]. To verify if GAs had any hemolytic activity, their effect on human red blood cells (hRBC) was tested. Different fractions containing GAs were spot tested on tryptic soy agar containing RBC (5% hRBC). Results in Fig. 8B (left panel) indicated that GAs, their parent fraction #194 or *G. sylvestre* extract (GE) did not lyse hRBC. Positive controls including *Staphylococcus aureus* growth or PBS containing Triton X-100 caused clear halos around the spots. While *S. aureus* mediated clearance is due to its secreted hemolytic activity, Triton X-100 disrupts cell membranes by its detergent activity. Cytotoxicity of GAs towards monolayers of human intestinal epithelial cells (Int-407, data not included) and African green monkey kidney cells was also evaluated. This did not reveal any significant difference between mock treated and GAs (40 µg/ml) treated cells suggesting that GAs are nontoxic to the mammalian cells used in this study (Fig. 8B, right panels).

In summary, we have shown that GAs are nontoxic molecules to worms, mammalian cells and yeasts, and potent inhibitors of the yeast-to-hypha transition and hyphal growth in *C. albicans*, thus preventing pathogenesis in a non-mammalian model of Candida infection. Additional results indicate that GAs can prevent biofilm formation by *C. albicans* (data not included), possibly owing to their ability to inhibit hyphal morphogenesis that is central to this process. Moreover, GAs inhibit the growth of filamentous fungi of the *Aspergillus* genus. Hence, GAs might prove useful in the development of antifungal therapies targeting a key virulence attribute of *C. albicans* and other fungal pathogens. Interestingly, GAs inhibition of *C. albicans* morphogenesis was retained when assayed in serum-containing medium suggesting that GAs were not depleted or rendered ineffective by serum components (Fig. 4). GAs might have several targets along the regulatory pathway involved in the expression of hypha-specific genes or in a pathway that is necessary for hyphal morphogenesis under several hypha-inducing conditions. Our observation of GA-treated yeast cells revealed the accumulation of vesicles (see Fig. 3) suggesting that GAs might in particular alter vacuolar function that is required for efficient hyphal differentiation [53]. Yet, further experiments such as transcript profiling, fitness profiling using collections of knock-out or over-expression mutants in *S. cerevisiae* or *C. albicans* [54], [55], [56], [57], and target purification by affinity will be needed to precisely decipher the target(s) of GAs. Identifying this(these) target(s) might trigger the discovery of additional inhibitors of fungal morphogenesis with broader applicability than GAs whose triterpenoid saponin core structure is complex for synthesis to generate a structure-activity relation for the improvement of bioactivity.

## Supporting Information

Figure S1 MS and analytical chromatograms of GA-IV (**2**) as detected by ELSD, MS ES+ TIC and MS ES- TIC mode, respectively.
(PDF)

Figure S2 Low Resolution Mass spectra of GA-IV (**2**) (ESI+).
(PDF)

Figure S3 High Resolution Mass spectra of GA-IV (**2**) (ESI+).
(PDF)

Figure S4 ^1^H NMR spectra of GA-IV (**2**) in C_5_D_5_N (600 MHz).
(PDF)

Figure S5 ^13^C NMR spectra of GA-IV (**2**) in C_5_D_5_N (150 MHz).
(PDF)

Figure S6 MS and analytical chromatograms of GA-III (**1**) as detected by ELSD, MS ES+ TIC and MS ES- TIC mode, respectively.
(PDF)

Figure S7 Low Resolution Mass spectra of GA-III (**1**) (ESI+).
(PDF)

Figure S8 High Resolution Mass spectra of GA-III (**1**) (ESI+).
(PDF)

Figure S9 ^1^H NMR spectra of GA-III (**1**) in C_5_D_5_N (600 MHz).
(PDF)

Figure S10 ^13^C NMR spectra of GA-III (**1**) in C_5_D_5_N (150 MHz).
(PDF)

Figure S11 MS and analytical chromatograms of GA-XIV (**4**) as detected by ELSD, MS ES+ TIC and MS ES- TIC mode, respectively.
(PDF)

Figure S12 Low Resolution Mass spectra of GA-XIV (**4**) (ESI+).
(PDF)

Figure S13 High Resolution Mass spectra of GA-XIV (**4**) (ESI+).
(PDF)

Figure S14 ^13^H NMR spectra of GA-XIV (**4**) in C_5_D_5_N (600 MHz).
(PDF)

Figure S15 ^13^C NMR spectra of GA-XIV (**4**) in C_5_D_5_N (150 MHz).
(PDF)

Figure S16 MS and analytical chromatograms of GA-XIII (**3**) as detected by ELSD, MS ES+ TIC and MS ES- TIC mode, respectively.
(PDF)

Figure S17 Low Resolution Mass spectra of GA-XIII (**3**) (ESI+).
(PDF)

Figure S18 High Resolution Mass spectra of GA-XIII (**3**) (ESI+).
(PDF)

Figure S19 ^13^H NMR spectra of GA-XIII (**3**) in C_5_D_5_N (600 MHz).
(PDF)

Figure S20 ^13^C NMR spectra of GA-XIII (**3**) in C_5_D_5_N (150 MHz).
(PDF)

Figure S21 GAs inhibit *C. albicans* yeast-to-hyphal transition in other hyphal growth conditions.
(PDF)
