# Supplementary figures and images for: Gymnemic Acids Inhibit Hyphal Growth and Virulence in Candida albicans
Source: PLoS One. 2013 Sep 11;8(9):e74189. doi: 10.1371/journal.pone.0074189 (PMC3770570; doi:10.1371/journal.pone.0074189)

Figure S2. Low Resolution Mass spectra of GA-IV (2) (ESI+).

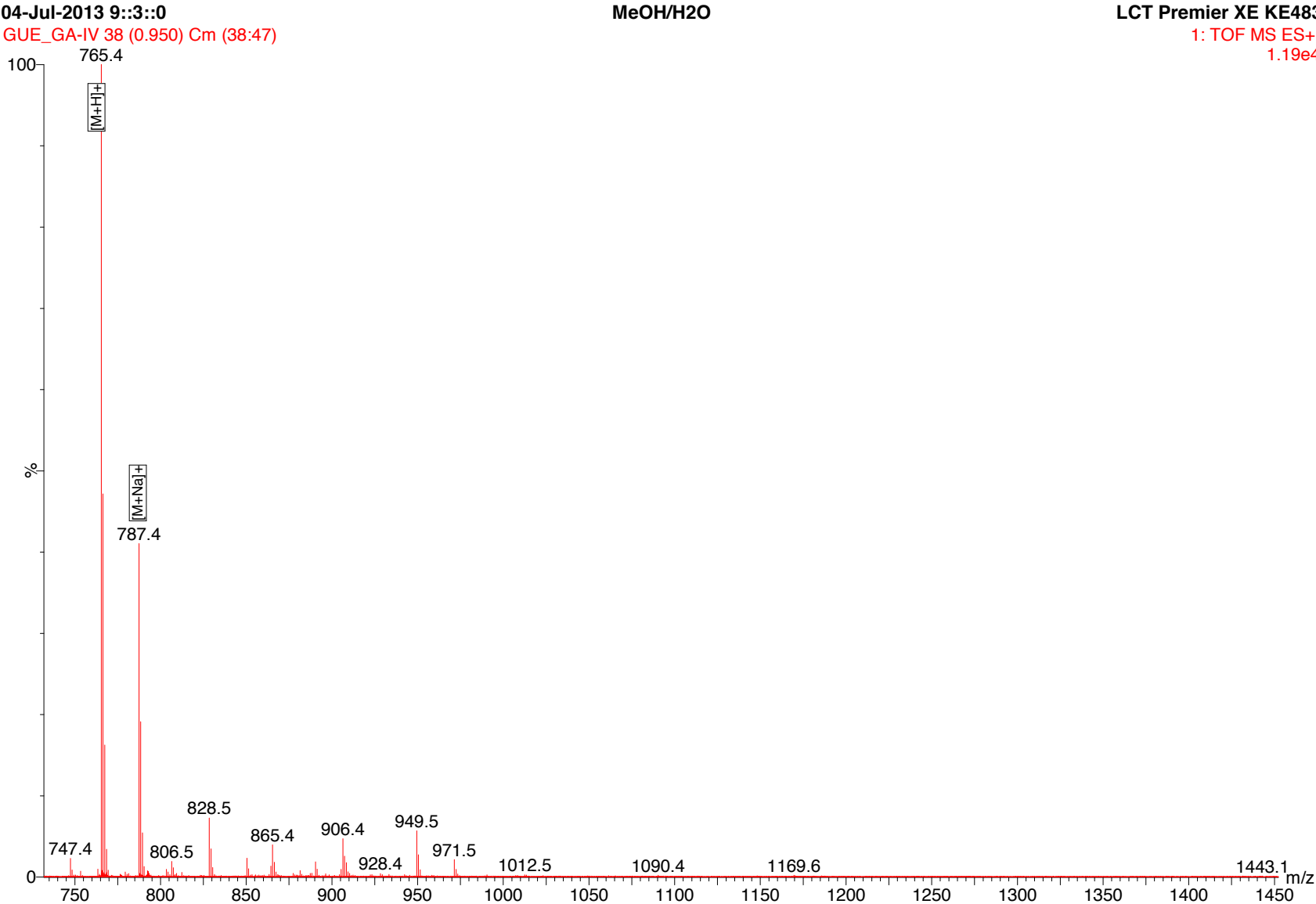

Supplement: Figure S2 — Low Resolution Mass spectra of GA-IV (2) (ESI+). (PDF) [file pone.0074189.s002.pdf]

Figure S4.  $^1\text{H}$  NMR spectra of GA-IV (**2**) in  $\text{C}_5\text{D}_5\text{N}$  (600 MHz).

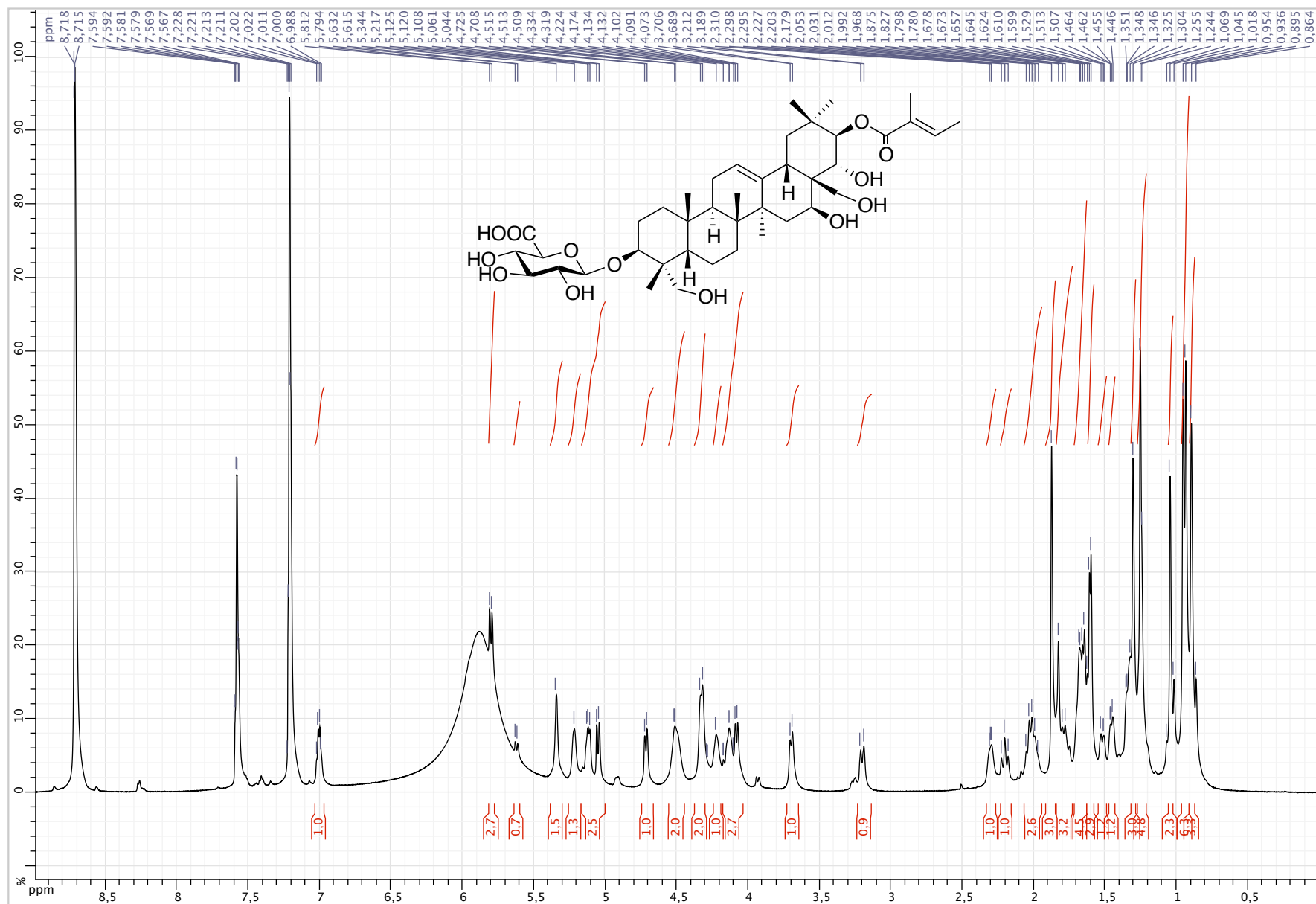

Supplement: Figure S4 — 1H NMR spectra of GA-IV (2) in C5D5N (600 MHz). (PDF) [file pone.0074189.s004.pdf]

Figure S5.  $^{13}\text{C}$  NMR spectra of GA-IV (**2**) in  $\text{C}_5\text{D}_5\text{N}$  (150 MHz).

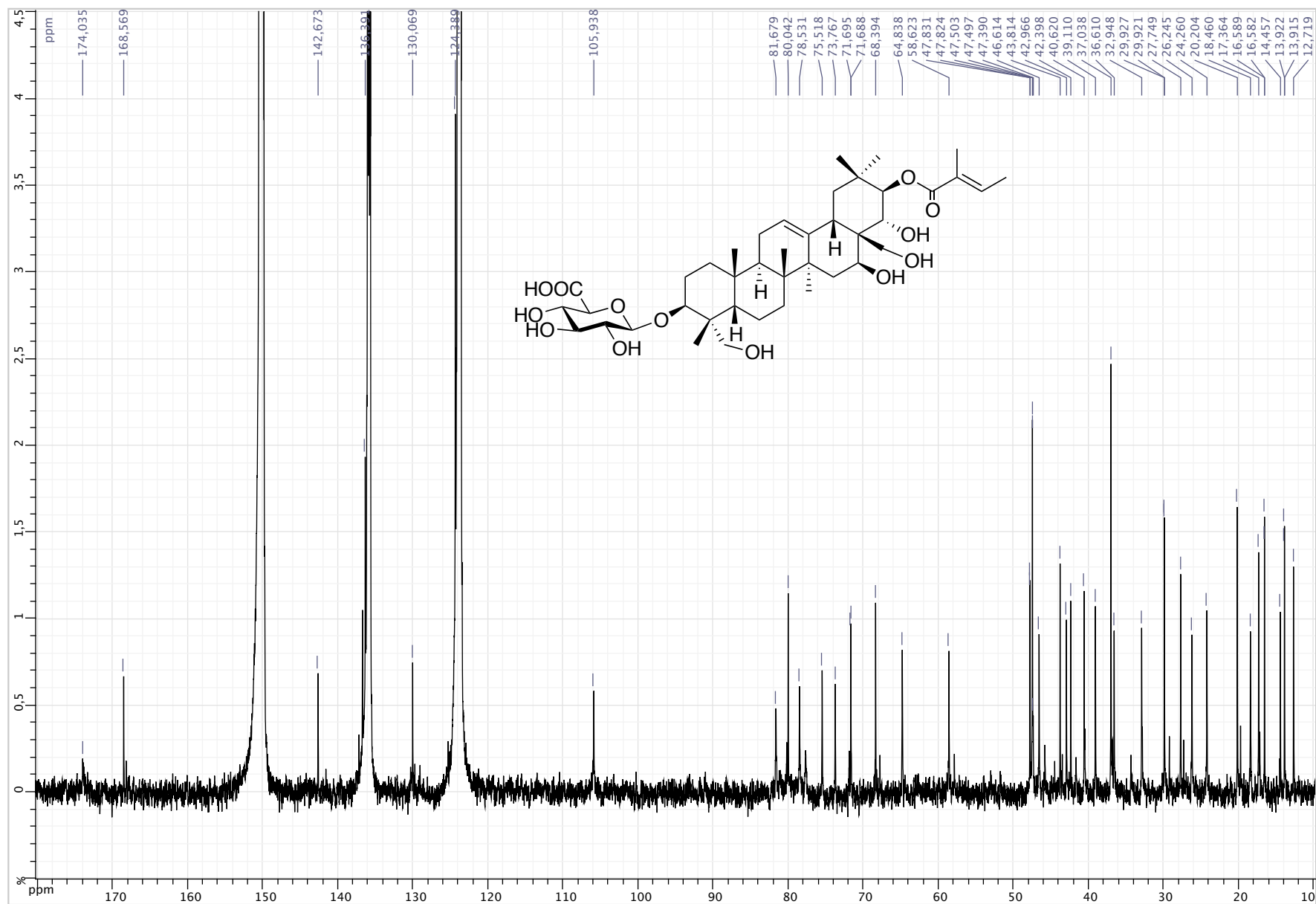

Supplement: Figure S5 — 13C NMR spectra of GA-IV (2) in C5D5N (150 MHz). (PDF) [file pone.0074189.s005.pdf]

Figure S7. Low Resolution Mass spectra of GA-III (1) (ESI+).

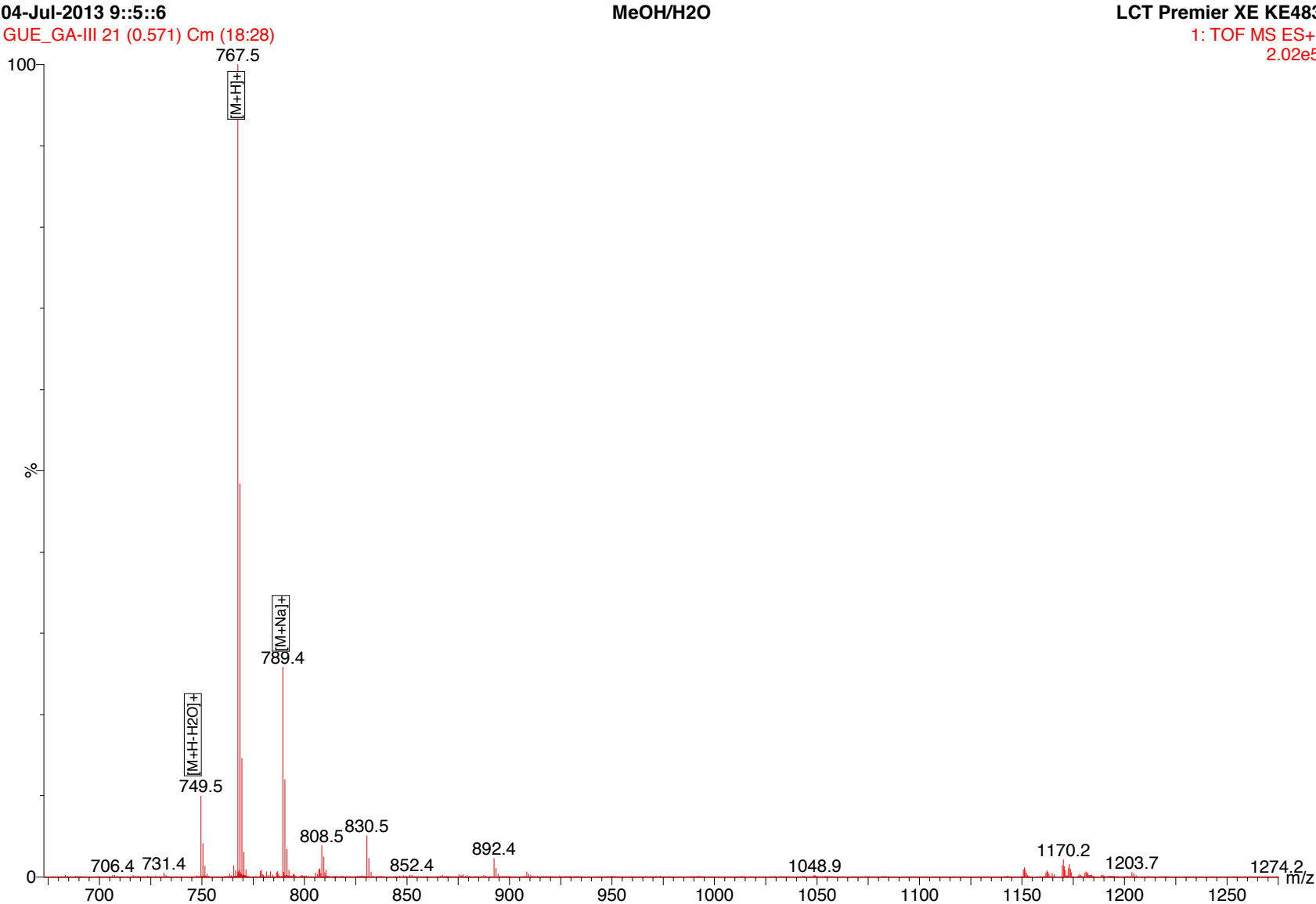

Supplement: Figure S7 — Low Resolution Mass spectra of GA-III (1) (ESI+). (PDF) [file pone.0074189.s007.pdf]

Figure S9.  $^1\text{H}$  NMR spectra of GA-III (**1**) in  $\text{C}_5\text{D}_5\text{N}$  (600 MHz).

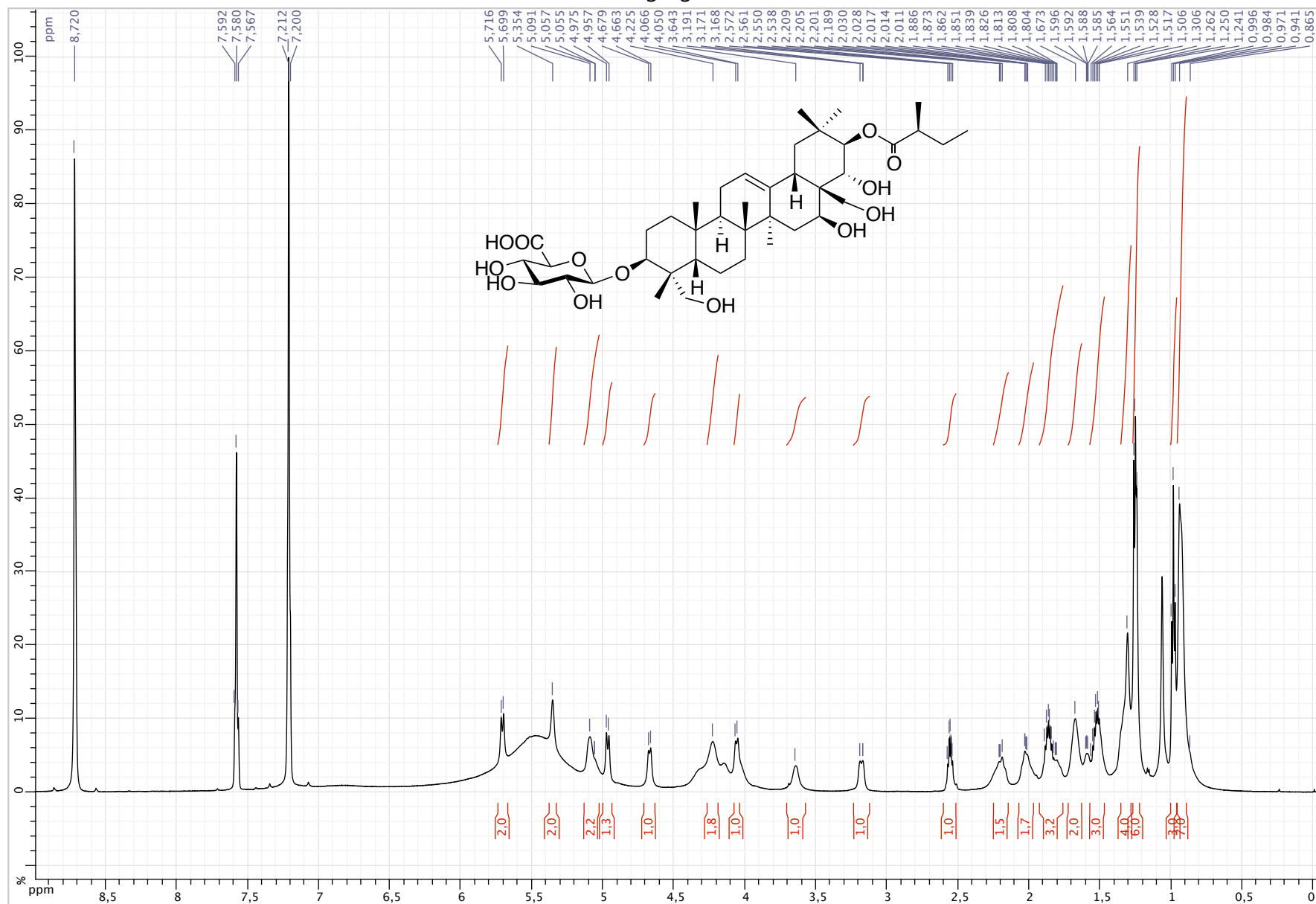

Supplement: Figure S9 — 1H NMR spectra of GA-III (1) in C5D5N (600 MHz). (PDF) [file pone.0074189.s009.pdf]

Figure S10.  $^{13}\text{C}$  NMR spectra of GA-III (**1**) in  $\text{C}_5\text{D}_5\text{N}$  (150 MHz).

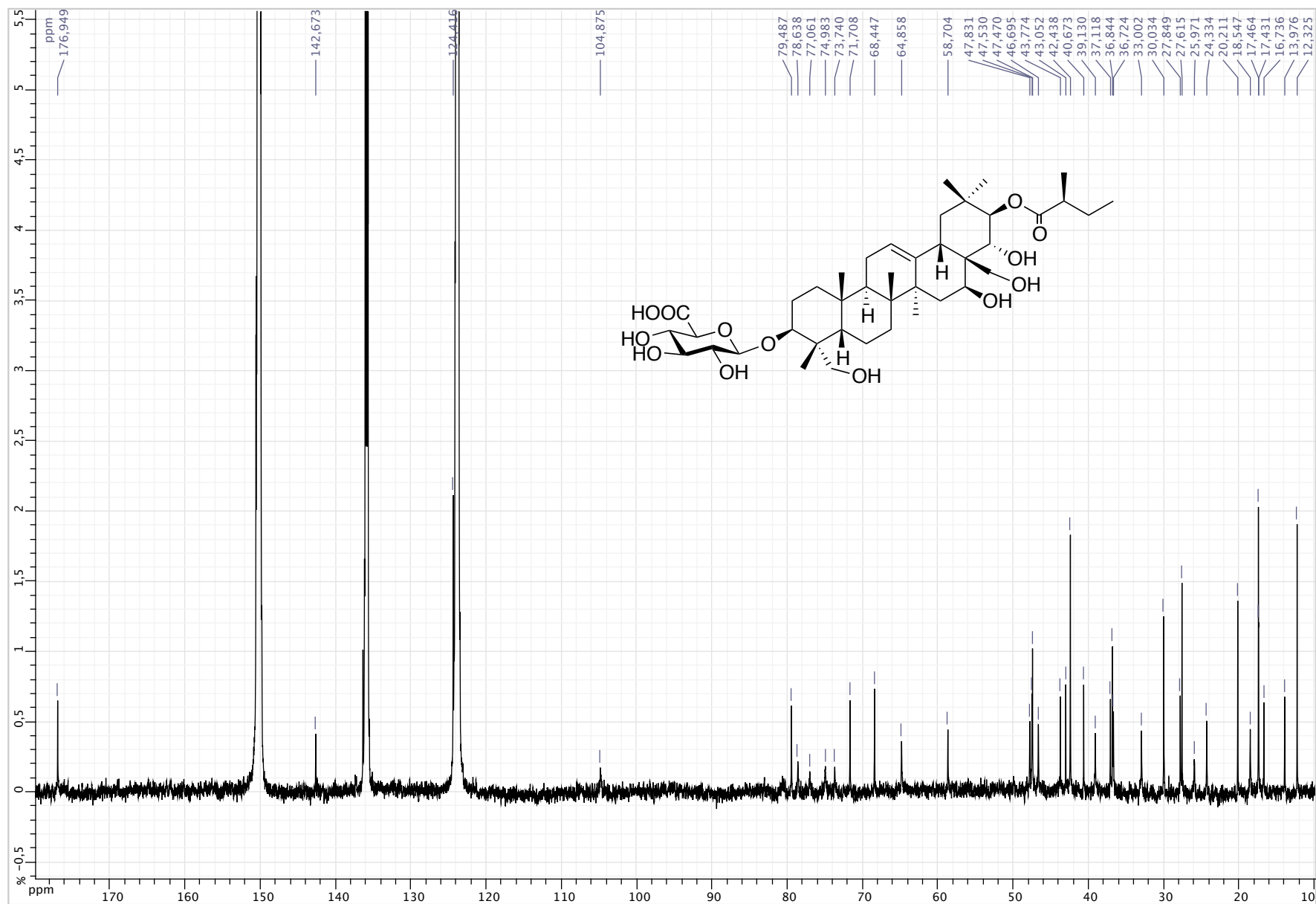

Supplement: Figure S10 — 13C NMR spectra of GA-III (1) in C5D5N (150 MHz). (PDF) [file pone.0074189.s010.pdf]

Figure S12. Low Resolution Mass spectra of GA-XIV (4) (ESI+).

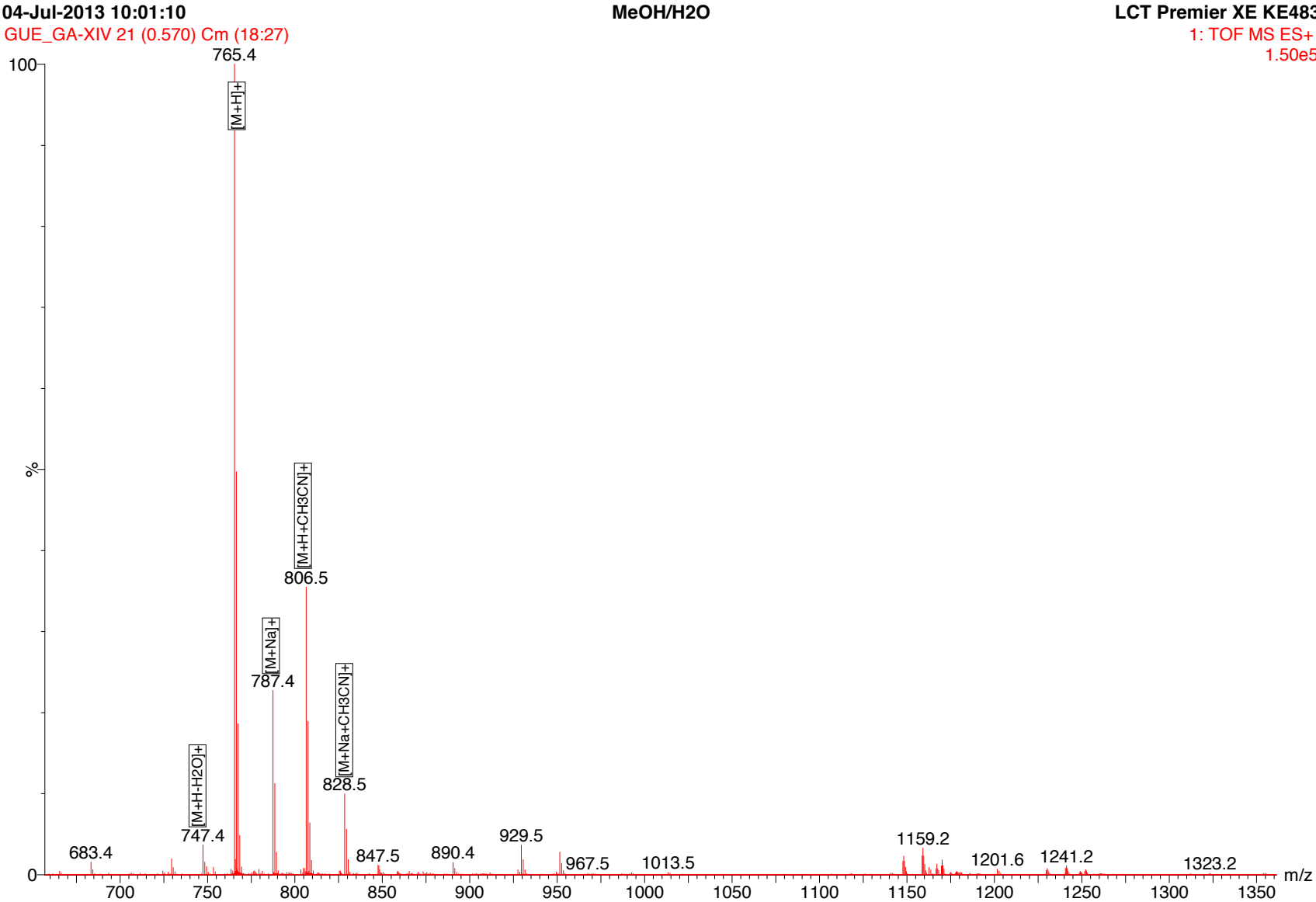

Supplement: Figure S12 — Low Resolution Mass spectra of GA-XIV (4) (ESI+). (PDF) [file pone.0074189.s012.pdf]

Figure S14.  $^1\text{H}$  NMR spectra of GA-XIV (**4**) in  $\text{C}_5\text{D}_5\text{N}$  (600 MHz).

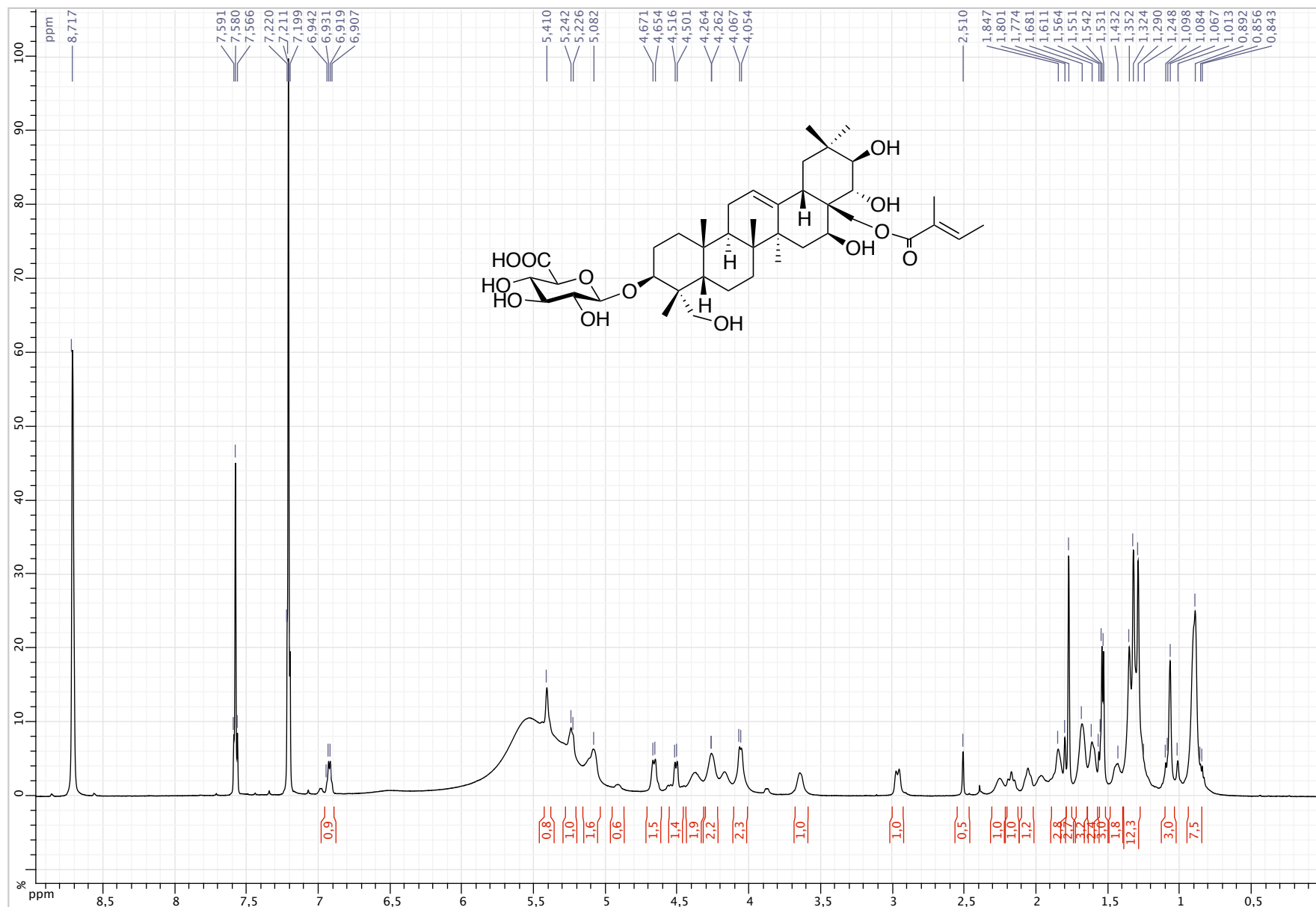

Supplement: Figure S14 — 13H NMR spectra of GA-XIV (4) in C5D5N (600 MHz). (PDF) [file pone.0074189.s014.pdf]

Figure S15.  $^{13}\text{C}$  NMR spectra of GA-XIV (**4**) in  $\text{C}_5\text{D}_5\text{N}$  (150 MHz).

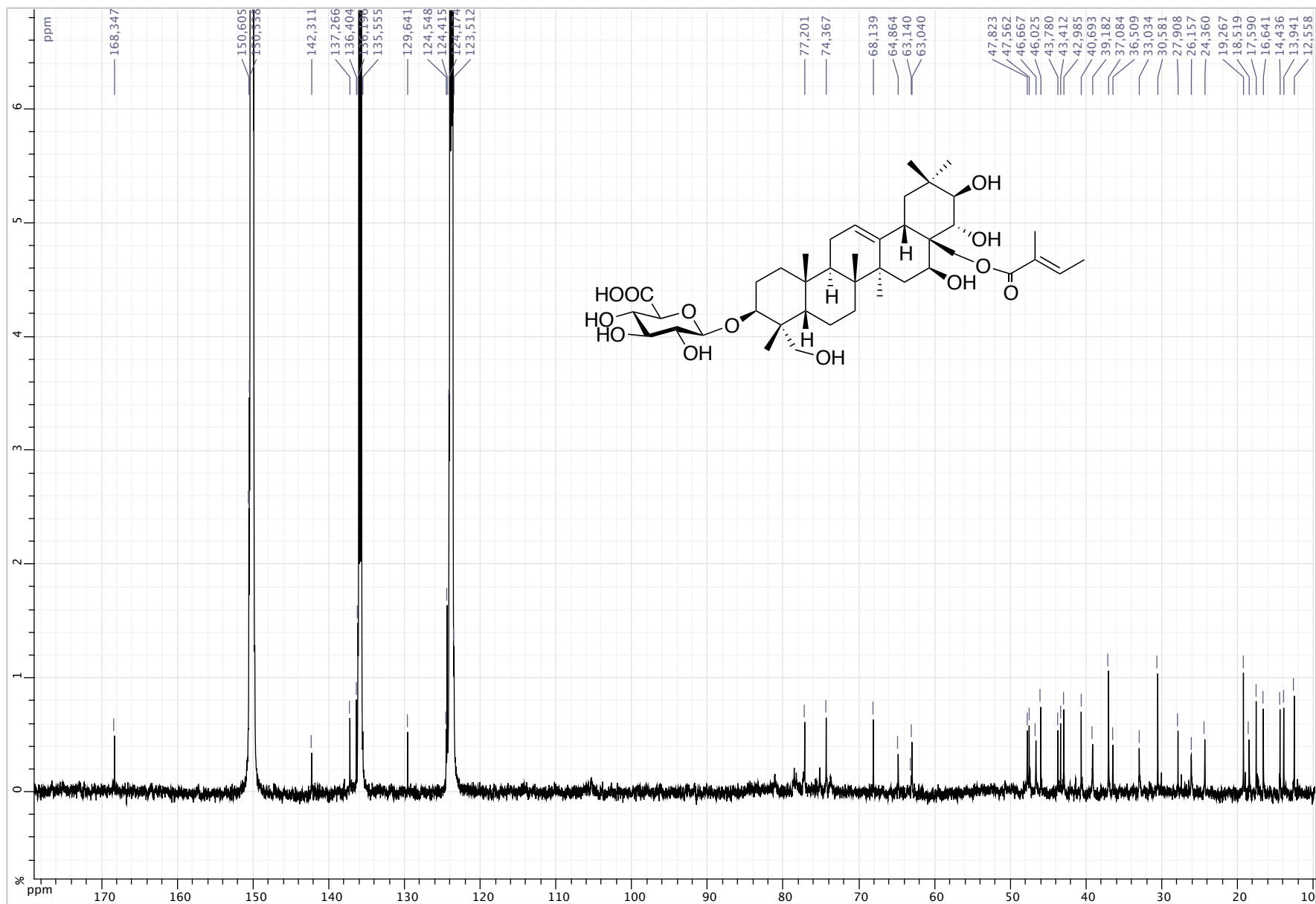

Supplement: Figure S15 — 13C NMR spectra of GA-XIV (4) in C5D5N (150 MHz). (PDF) [file pone.0074189.s015.pdf]

Figure S17. Low Resolution Mass spectra of GA-XIII (3) (ESI+).

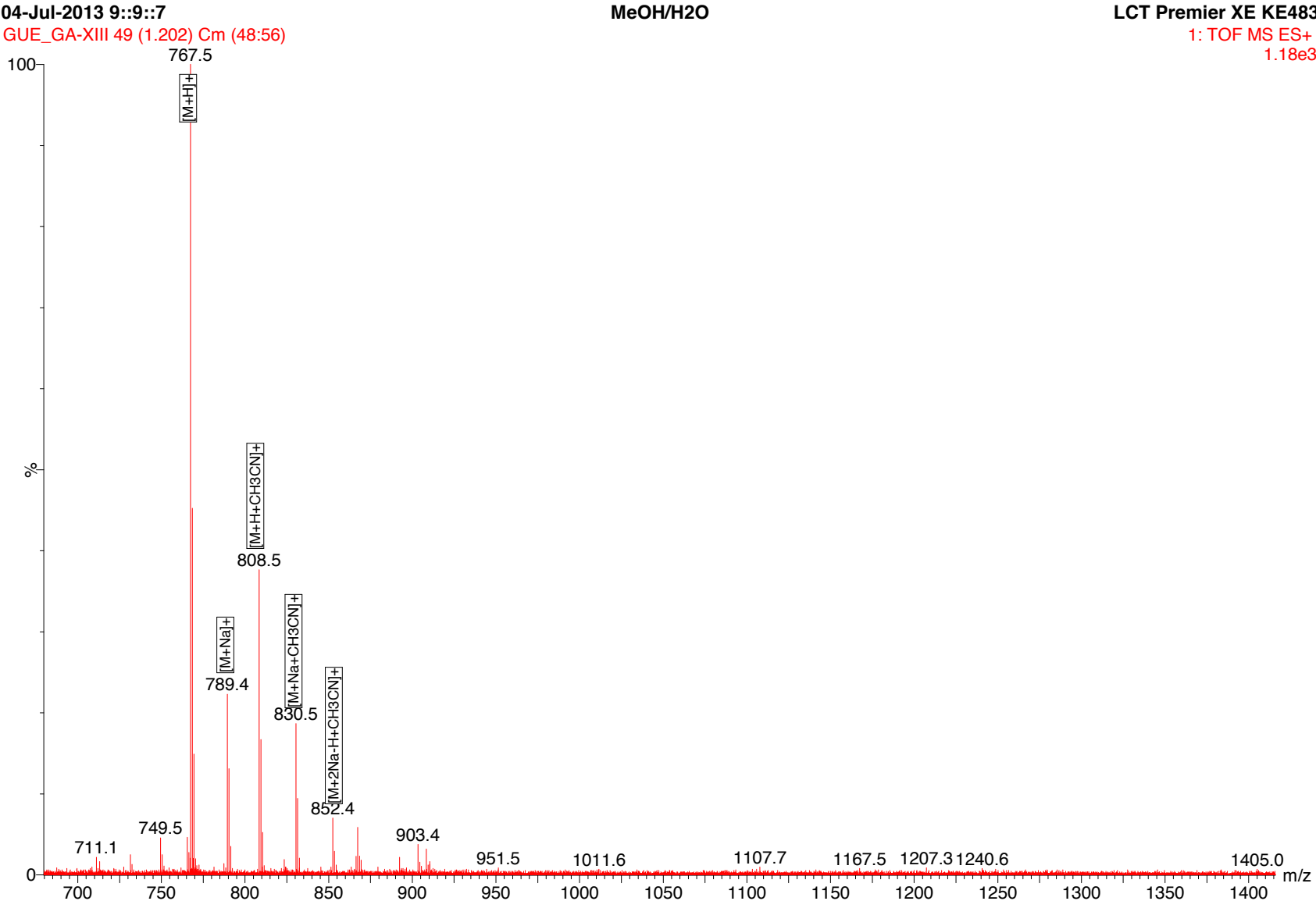

Supplement: Figure S17 — Low Resolution Mass spectra of GA-XIII (3) (ESI+). (PDF) [file pone.0074189.s017.pdf]

Figure S20.  $^{13}\text{C}$  NMR spectra of GA-XIII (**3**) (150 MHz).

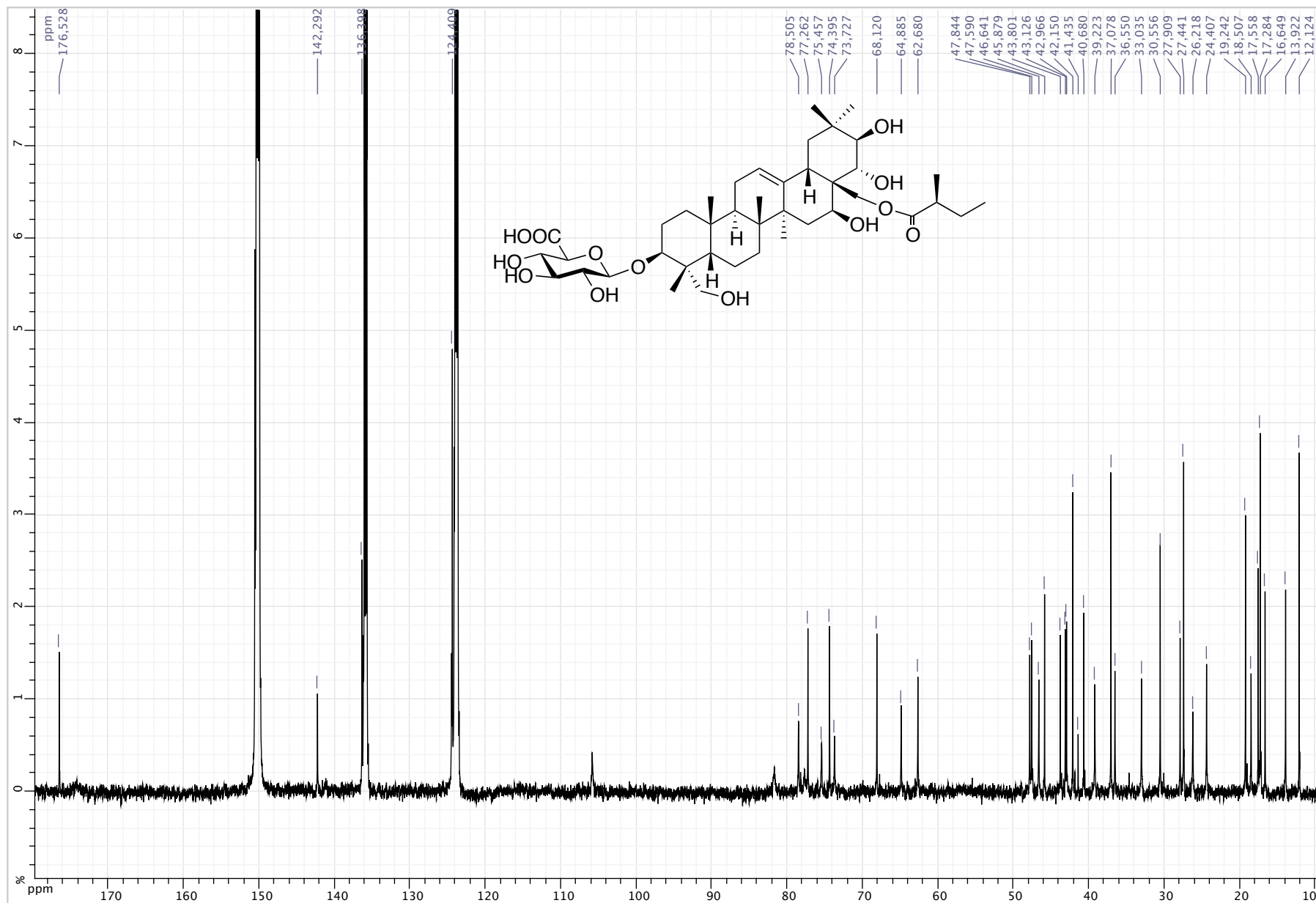

Supplement: Figure S20 — 13C NMR spectra of GA-XIII (3) in C5D5N (150 MHz). (PDF) [file pone.0074189.s020.pdf]

Figure S21. GAs inhibit *C. albicans* yeast-to-hyphal transition in other hyphal growth conditions.

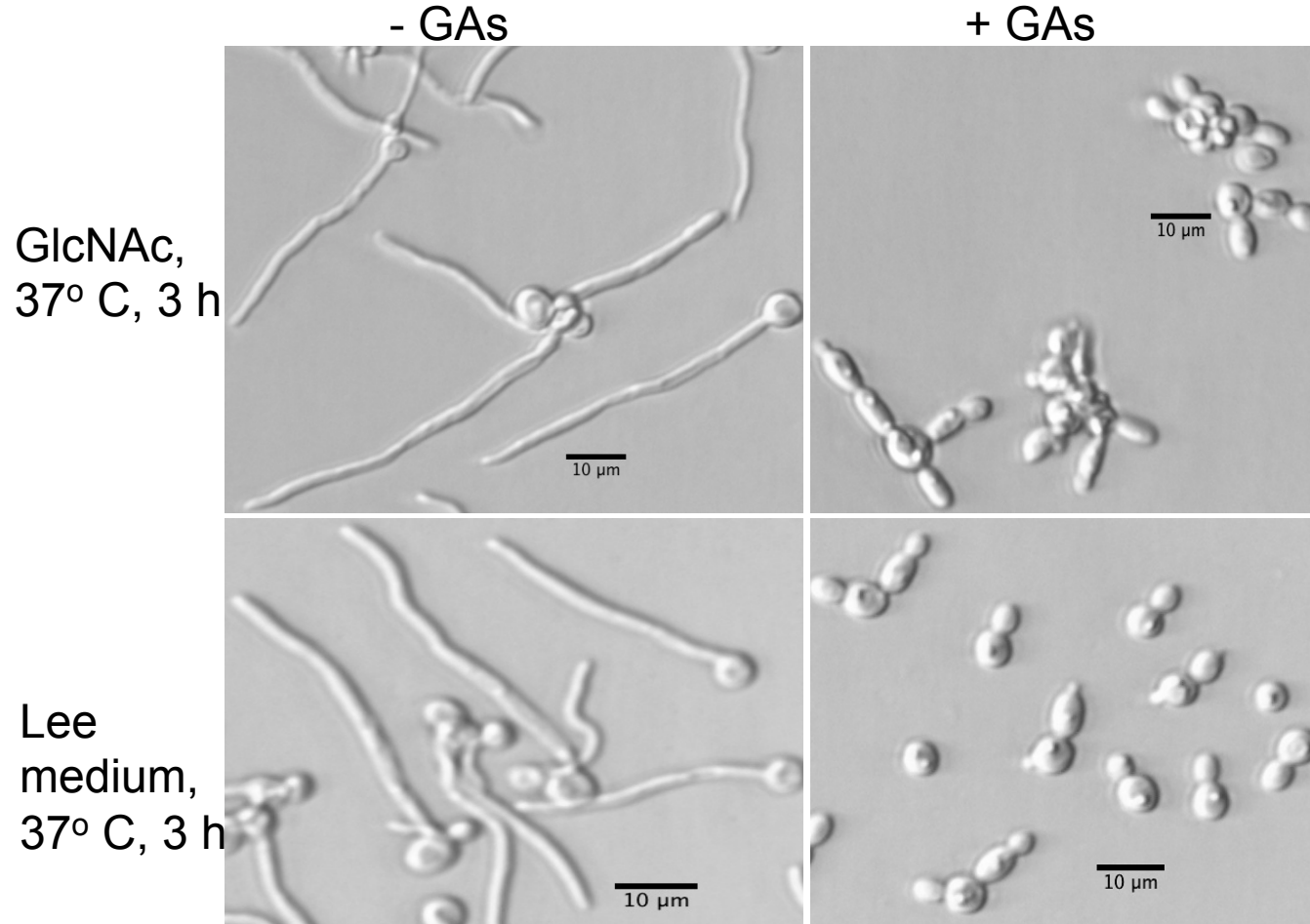

Supplement: Figure S21 — GAs inhibit C. albicans yeast-to-hyphal transition in other hyphal growth conditions. (PDF) [file pone.0074189.s021.pdf]
