# Supplementary material for: Gymnemic Acids Inhibit Hyphal Growth and Virulence in Candida albicans
Source: PLoS One. 2013 Sep 11;8(9):e74189. doi: 10.1371/journal.pone.0074189 (PMC3770570; doi:10.1371/journal.pone.0074189)

Figure S6. MS and analytical chromatograms of GA-III (**1**) as detected by ELSD, MS ES+ TIC and MS ES- TIC mode, respectively.

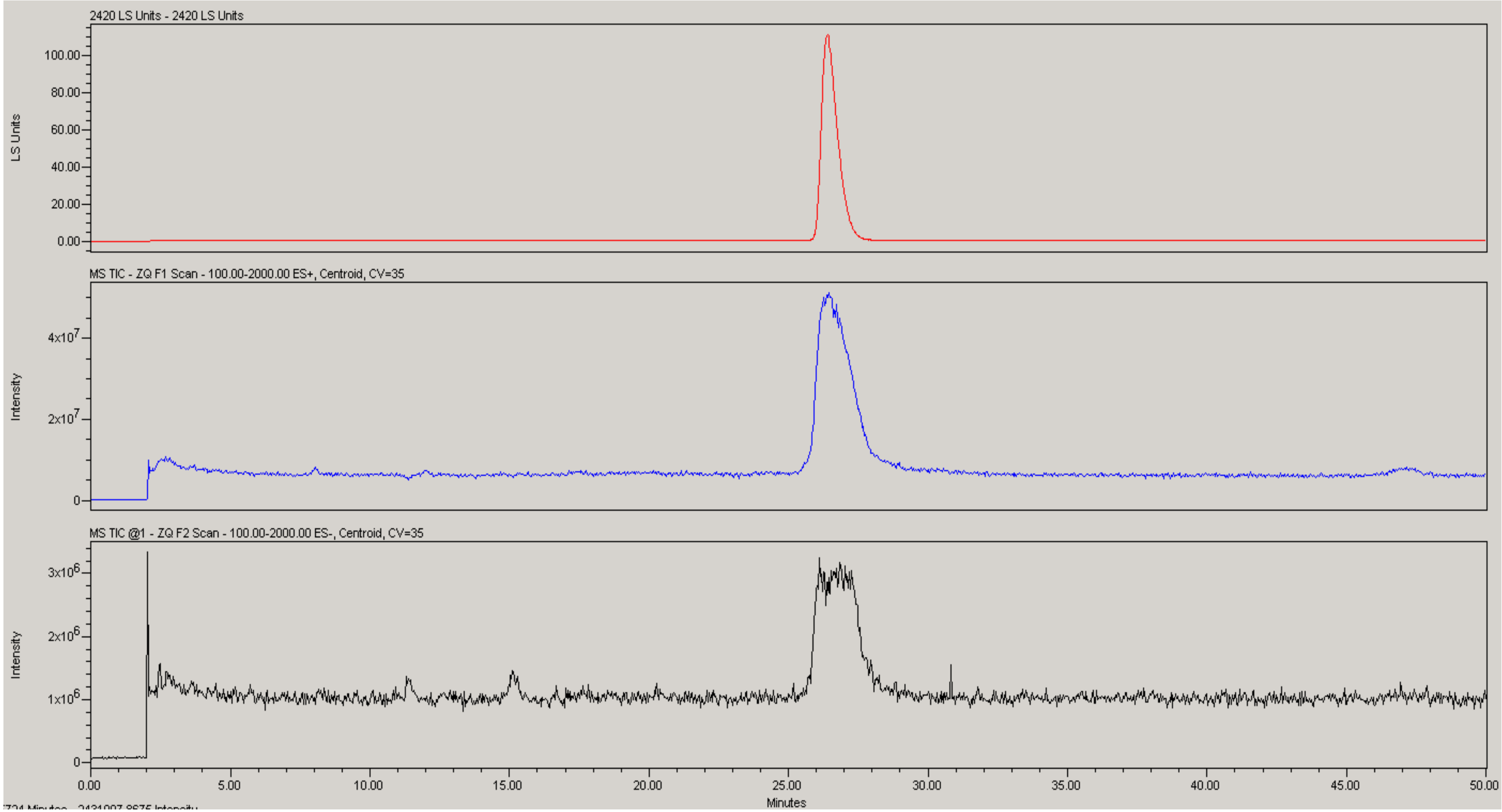

Supplement: Figure S6 — MS and analytical chromatograms of GA-III (1) as detected by ELSD, MS ES+ TIC and MS ES- TIC mode, respectively. (PDF) [file pone.0074189.s006.pdf]
