# Supplementary material for: Gymnemic Acids Inhibit Hyphal Growth and Virulence in Candida albicans
Source: PLoS One. 2013 Sep 11;8(9):e74189. doi: 10.1371/journal.pone.0074189 (PMC3770570; doi:10.1371/journal.pone.0074189)
Supplement: Figure S8 — High Resolution Mass spectra of GA-III (1) (ESI+). (PDF) [file pone.0074189.s008.pdf]

Figure S8. High Resolution Mass spectra of GA-III (**1**) (ESI+).

Elemental Composition Report

Single Mass Analysis
Tolerance = 5.0 PPM / DBE: min = -1.5, max = 100.0
Element prediction: Off
Number of isotope peaks used for i-FIT = 9

Monoisotopic Mass, Even Electron Ions
136 formula(e) evaluated with 1 results within limits (all results (up to 1000) for each mass)
Elements Used:
C: 1-150 H: 1-150 O: 0-15

04-Jul-2013 9::5::6 MeOH/H2O LCT Premier XE KE483
GUE_GA-III 53 (1.293) Cm (49:59) 1: TOF MS ES+
8.45e+002

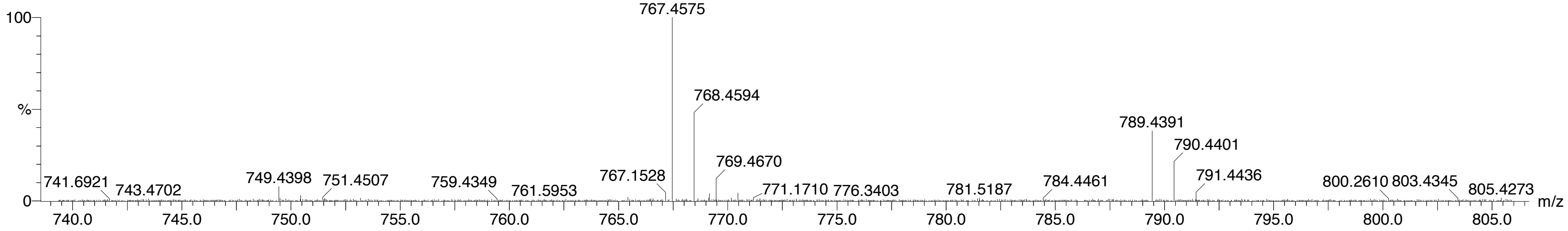

Minimum:				-1.5			
Maximum:		5.0	5.0	100.0			
Mass	Calc. Mass	mDa	PPM	DBE	i-FIT	i-FIT (Norm)	Formula
767.4575	767.4582	-0.7	-0.9	8.5	387.0	0.0	C41 H67 O13 $[M+H]^+$
