# Supplementary material for: Gymnemic Acids Inhibit Hyphal Growth and Virulence in Candida albicans
Source: PLoS One. 2013 Sep 11;8(9):e74189. doi: 10.1371/journal.pone.0074189 (PMC3770570; doi:10.1371/journal.pone.0074189)

Figure S11. MS and analytical chromatograms of GA-XIV (**4**) as detected by ELSD, MS ES+ TIC and MS ES- TIC mode, respectively.

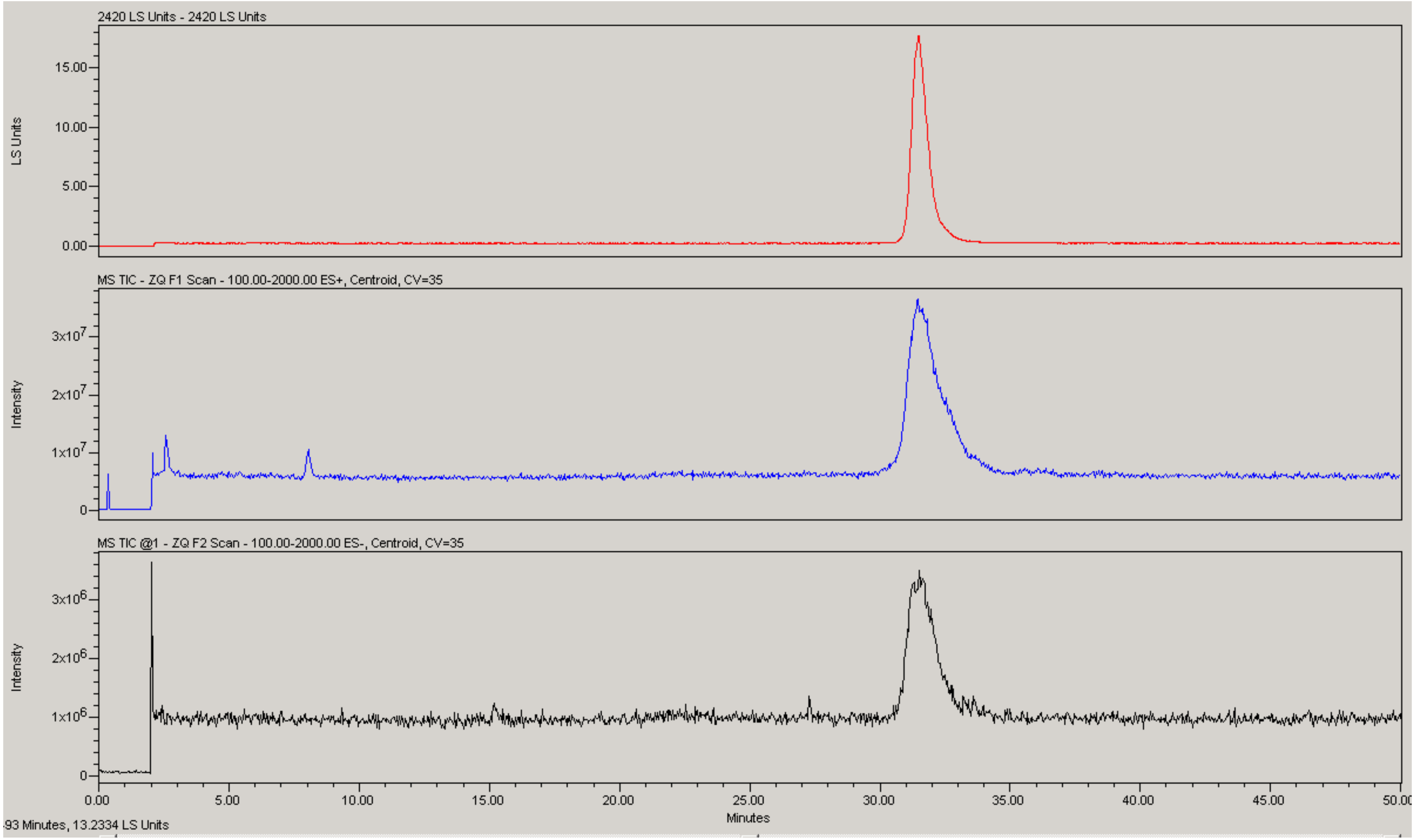

Supplement: Figure S11 — MS and analytical chromatograms of GA-XIV (4) as detected by ELSD, MS ES+ TIC and MS ES- TIC mode, respectively. (PDF) [file pone.0074189.s011.pdf]
