# Supplementary material for: Gymnemic Acids Inhibit Hyphal Growth and Virulence in Candida albicans
Source: PLoS One. 2013 Sep 11;8(9):e74189. doi: 10.1371/journal.pone.0074189 (PMC3770570; doi:10.1371/journal.pone.0074189)
Supplement: Figure S13 — High Resolution Mass spectra of GA-XIV (4) (ESI+). (PDF) [file pone.0074189.s013.pdf]

Figure S13. High Resolution Mass spectra of GA-XIV (**4**) (ESI+).

Elemental Composition Report

Single Mass Analysis
Tolerance = 5.0 PPM / DBE: min = -1.5, max = 100.0
Element prediction: Off
Number of isotope peaks used for i-FIT = 9

Monoisotopic Mass, Even Electron Ions
138 formula(e) evaluated with 2 results within limits (all results (up to 1000) for each mass)
Elements Used:
C: 1-150 H: 1-150 O: 0-15

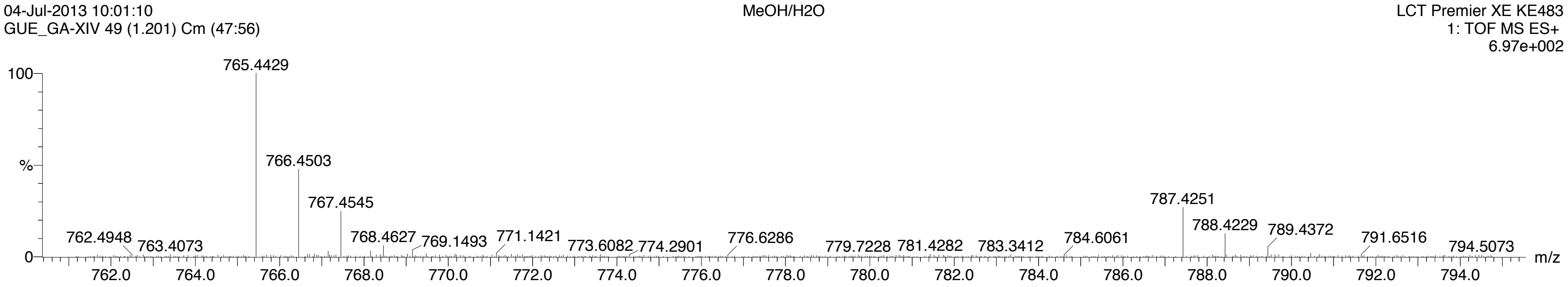

Mass	Calc. Mass	mDa	PPM	DBE	i-FIT	i-FIT (Norm)	Formula
Minimum:				-1.5			
Maximum:		5.0	5.0	100.0			
765.4429	765.4460	-3.1	-4.0	31.5	387.6	0.1	C59 H57
	765.4425	0.4	0.5	9.5	389.8	2.3	C41 H65 O13

$[M+H]^+$
