# Supplementary material for: Gymnemic Acids Inhibit Hyphal Growth and Virulence in Candida albicans
Source: PLoS One. 2013 Sep 11;8(9):e74189. doi: 10.1371/journal.pone.0074189 (PMC3770570; doi:10.1371/journal.pone.0074189)

Figure S19. ^{1}H NMR spectra of GA-XIII (**3**) in C_5D_5N (600 MHz).

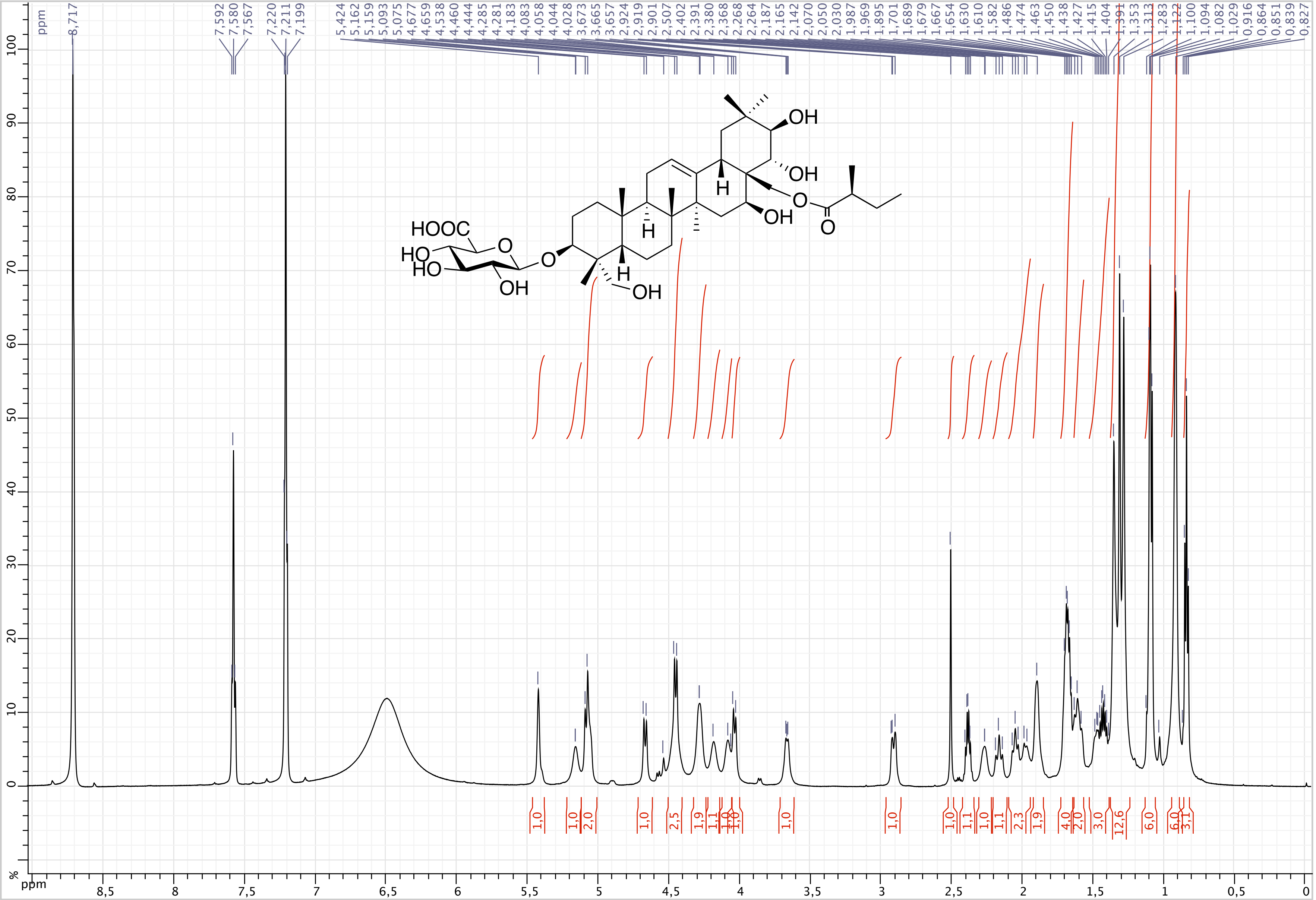

Supplement: Figure S19 — 13H NMR spectra of GA-XIII (3) in C5D5N (600 MHz). (PDF) [file pone.0074189.s019.pdf]
